# Mud and burnt Roman bricks from Romula

**DOI:** 10.1038/s41598-022-19427-7

**Published:** 2022-09-23

**Authors:** P. Badica, A. Alexandru-Dinu, M. A. Grigoroscuta, M. Burdusel, G. V. Aldica, V. Sandu, C. Bartha, S. Polosan, A. Galatanu, V. Kuncser, M. Enculescu, C. Locovei, I. Porosnicu, I. Tiseanu, M. Ferbinteanu, I. Savulescu, M. Negru, N. D. Batalu

**Affiliations:** 1 National Institute of Materials Physics, Atomistilor Street 405A, 077125 Magurele, Romania; 2 Faculty of Physics, University of Bucharest, Atomistilor Street 405, 077125 Magurele, Romania; 3 National Institute of Laser, Radiation, and Plasma Physics, Street Atomistilor 409, 077125 Magurele, Romania; 4 Faculty of Chemistry, University of Bucharest, Panduri Road 90, 050663 Bucharest, Romania; 5 Faculty of Geography, University of Bucharest, Bd. Nicolae Balcescu 1, Bucharest, Romania; 6 CICSA, Faculty of History, University of Bucharest, Bd. Regina Maria 4-12, Bucharest, Romania; 7 Faculty of Juridical and Administrative Sciences, Spiru Haret University Bucharest, Bd. Soseaua Berceni 24, 400123 Bucharest, Romania; 8 University of Craiova, Faculty of Social Sciences, A. I. Cuza 13, Craiova, Romania; 9 University Politehnica of Bucharest, Splaiul Independentei 313, 060042 Bucharest, Romania

**Keywords:** Ceramics, Archaeology

## Abstract

Sesquipedalian mud and burnt bricks (second to third century AD) were excavated from the Roman city of Romula located in the Lower Danube Region (Olt county, Romania). Along with local soils, bricks are investigated by petrographic analysis, X-ray fluorescence (XRF), X-ray diffraction (XRD), Fourier transformed infrared spectroscopy (FT-IR), electron microscopy (SEM/EDX), X-ray microtomography (XRT), thermal analysis (DTA-TG), Mӧssbauer spectroscopy, magnetometry, colorimetry, and mechanical properties assessment. The results correlate well with each other, being useful for conservation/restoration purposes and as reference data for other ceramic materials. Remarkably, our analysis and comparison with literature data indicate possible control and wise optimization by the ancient brickmakers through the recipe, design (size, shape, and micro/macrostructure), and technology of the desired physical–chemical–mechanical properties. We discuss the Roman bricks as materials that can adapt to external factors, similar, to some extent, to modern “smart” or “intelligent” materials. These features can explain their outstanding durability to changes of weather/climate and mechanical load.

## Introduction

Bricks are old building materials (~ 8000 BC for mudbricks and ~ 3000 BC for burnt bricks, Mesopotamia^1,2^) and they are still in use nowadays, mostly for facades and walls between structural concrete elements, but on a significantly lower scale than in the ancient times when buildings were entirely made of bricks. This may induce the idea that the ancient bricks might be viewed as obsolete and deprecated. This is not the case since the ancient bricks proved to intrinsically incorporate modern concepts such as sustainability, durability, green and environment friendly materials and buildings, materials reuse/recycling. Therefore, ancient bricks may unveil unexpected know-how forgotten technologies and concepts.

Studies of ancient bricks from different periods, geographical locations, and cultures can also provide other valuable information: on one side the knowledge about ancient bricks and technologies is needed for appropriate restoration and conservation of the heritage constructions, and on the other side it can promote a better understanding of the regional development and society. Bricks through their archaeological availability and spreading, while being typically specific for the local production can be also viewed as convenient reference ceramic materials to compare with. For example, other ceramic artefacts such as amphorae, kitchen and storage ware are prone to trade activities, and comparison with local brick materials can provide and demonstrate different details of the commercial routes as well as other aspects of daily life. To do so, investigations of the ancient ceramic materials, including the bricks, are required.

**Figure 1:**
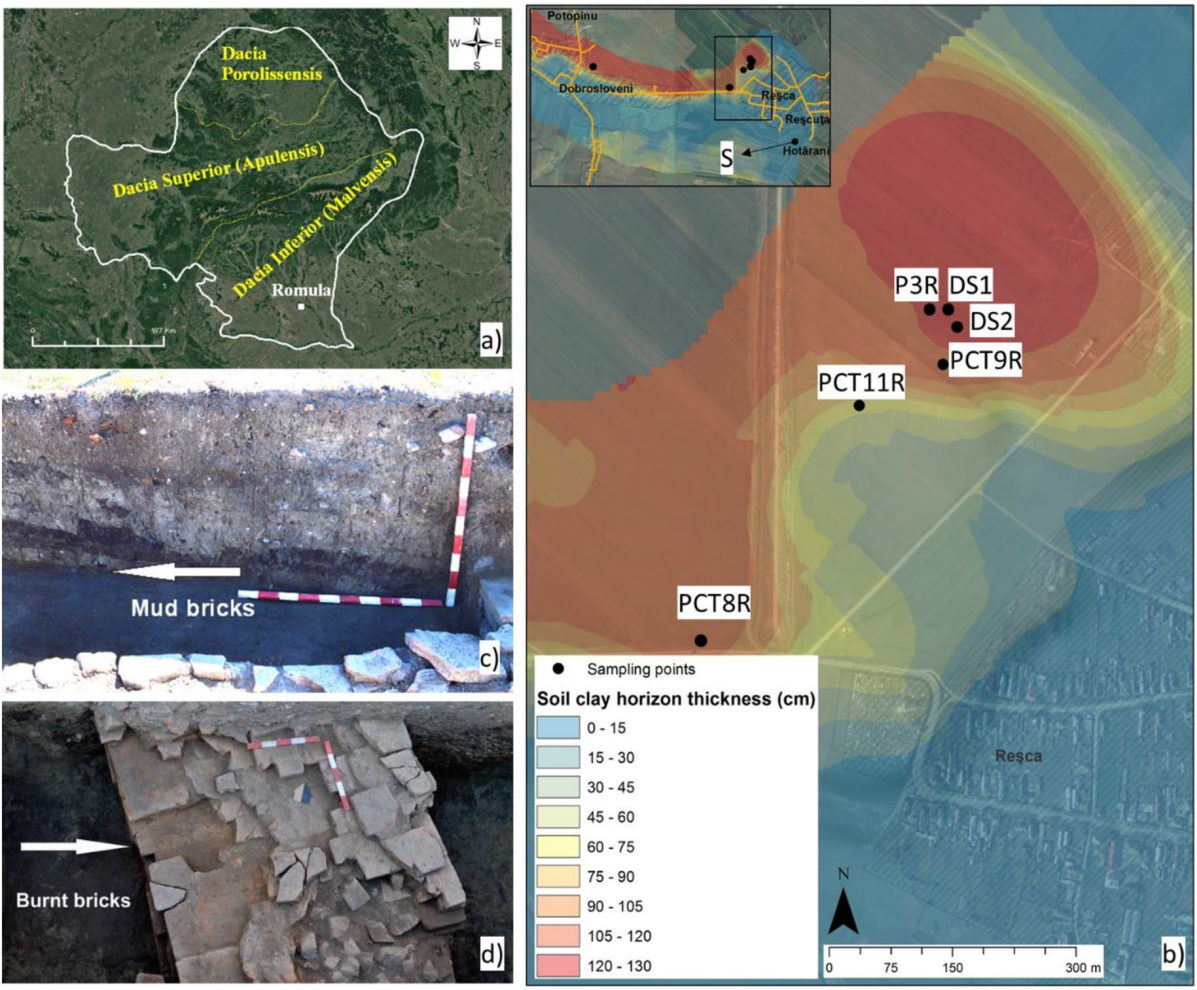
(**a,b**) Maps showing the location of the Romula city, and the type of soils. (**c**) The provenance of the ancient mudbrick from the enclosure wall of the first (central) Roman fortifications^9^ built in the first quarter of the second century AD. (**d**) The burnt brick from a wall built in the last decade of the second century to first third of the third century AD. For sample notation see Table 1. Map (**a**) was created using Google Earth (https://www.google.com/maps/@45.5432477,22.1410097,742653m/data=!3m1!1e3) on which Roman provinces were indicated. Map (**b**) was generated with ArcGIS v.10.5.

Romula was the largest Roman city in Dacia Inferior (Malvensis) (Fig. 1a) that played an important military, administrative, trade, production, and cultural role. The archaeological site of Romula (today Reșca village, the Olt County, Romania) covers about 3.06 km^2^ and it is the most extended site between the Carpathian Mountains and the Lower Danube River. Built by Romans as a fortress during the First War with Dacians (101–102 AD), Romula received the title of municipium (123–124 AD)^3^, and, later, of colonia (248 AD^4^, or in the time of Emperor Septimius Severus, 193–211 AD). It was abandoned in 271–275 AD in the time of Emperor Aurelian or even earlier, 253–268 AD, in the time of Emperor Gallienus. In the Northern Quarter were identified eight pottery workshops and 25 pottery kilns as evidence for an industrial-level fabrication of ceramic. Therefore, Romula was one of the largest productions centers of ceramic in the Lower Danube area, the discoveries of Roman ceramics being the reference for this region: the ceramic artefacts excavated in the site are building items (bricks, tiles, pavement pieces, and others), terracotta lamps, and pottery (amphorae, kitchen pottery, others)^5–7^. About 5 of the mentioned kilns were dedicated for the production of bricks and tiles and they were dated to the first decades of the third century AD^5,6^.

In this work, a complex archaeometric study of the representative mud and burnt sesquipedalian bricks found at Romula (second to third century AD) is performed. This is the first report on the advanced characterization of bricks from a Roman city located in the provinces of the Lower Danube. Sesquipedalian burnt brick can be compared directly with data from literature^8^ on similar bricks from other Roman provinces. Our work adds new information useful to propose refined correlations between different properties and a better understanding of Roman technology.

The work introduces in “Experimental”, samples, techniques, methodology and finite element approach used for the characterization of the local soils and of the mud and burnt bricks from Romula. This section is composed of two parts. The first one (“Samples of raw and archaeological materials”) presents the samples, their archaeological and geological context. The second part (“Samples physical characterization”) describes experimental characterization methods and simulation conditions. The fabrication in the laboratory of bulk samples by sintering at different temperatures of powders from a selected soil and from the mud brick is also described. “Results” is composed of five parts. Characterization of the local soils, mud and burnt bricks from Romula and their comparative analysis aiming at identification of the raw materials used for bricks fabrication are addressed in “Soils characterization and their identification as raw materials for fabrication of the ancient mud and burnt bricks”. In this subsection particle size analysis of the soils and data measured by X-ray diffraction, X-ray fluorescence, Fourier transformed infrared spectroscopy, thermal analysis and mass spectroscopy were used. “Porosity, water absorption, mechanical and thermal insulating properties of the burnt brick from Romula” is dedicated to the porosity, water absorption, mechanical and thermal insulating properties of the burnt brick from Romula. The traditional Archimedes method is complemented by observations through X-ray micro tomography. Mechanical properties are measured by standard compressive strength tests, and they are simulated by finite element analysis. Thermal insulating properties of the burnt brick are determined by flash calorimetry. In “Local structure and magnetic properties of materials from Romula” the fine structure of the soils and of the mud and burnt bricks as revealed by Mӧssbauer spectroscopy and magnetometry is presented. The as-obtained information related to Fe in compounds that are considered centres of colour is used and discussed versus the results of colorimetric measurements in “Colorimetric analysis of the soils and bricks from Romula”. The last part (“Microstructural aspects of the burnt brick from Romula”) is dedicated to microstructural characterization of the Roman burnt brick by electron microscopy. “Discussion” is structured into three parts. The first subsection (“Burning temperature of the Roman brick from Romula”) describes the deduction of the burning temperature. This is accomplished based on information from “Results” and on additional Vickers hardness data measured on the ancient burnt brick and on the bulk samples sintered in the laboratory at different temperatures from the investigated local soils that possibly are the raw materials. The second part (“Durability aspects of the Roman bricks”) discusses durability and response of the bricks to the environment based on our results and literature data. “Mechanical response aspects of the Roman burnt brick from Romula” focuses on mechanical adaptability under compressive load without the loss of integrity of the burnt brick, considering its specific structure, microstructure, and shape. The results are summarized in “Conclusion” emphasizing the criteria for the fabrication of durable Roman bricks.

## Experimental

### Samples of raw and archaeological materials

Three types of samples were investigated (Table 1).

**Table 1:** Samples, notation, appearance, and excavation details.

Sample	Notation	Appearence	Location (see Fig. 1b)
Burnt brick	B	Sintered bulk	Depth 0.40–0.60 m
Mudbrick	S1-1a	Powder compact	Northern sector of the site
	S1-1b		
	S1–2		
Sand	S	Powder	Depth 0.50–1.20 m
Raw soil 1	P8R	Powder	Depth 0.45–0.60 m
Raw soil 2	PCT11R	Powder	Depth 0.8–1.05 m
Raw soil 3	P13D	Powder	Depth 1.10 m
Raw soil 4	P3R	Powder	Depth 1.25–1.40 m
Raw soil 5	PCT9R, PCT9Rp	Powder	Depth 1.53 m
Raw soil 6	DS 1	Powder	Depth 2.5 m
Raw soil 7	DS 2	Powder	Depth 3.0 m
Burnt Mudbrick	S1-2*	Powder compact	–
Burnt raw soil 5	PCT9R*	Powder	–

PCT9Rp was extracted at approximately the same location and depth as PCT9R; (*) as-excavated powder samples ignited in laboratory at 880 °C in air with a heating rate of 200 °C/h, and a dwell time of 1 h. Two categories of soils were defined: low depth soils (< 1.53 m) and high depth soils (2.5 and 3 m).

Materials, such as clay and sand, as possible raw materials for fabrication of ancient bricks were extracted with a pedological probe in the northern area of the city, in the proximity of the industrial Northern Quarter and near the Teslui stream. The clay soil horizon is presented in Fig. 1.

**Figure 2:**
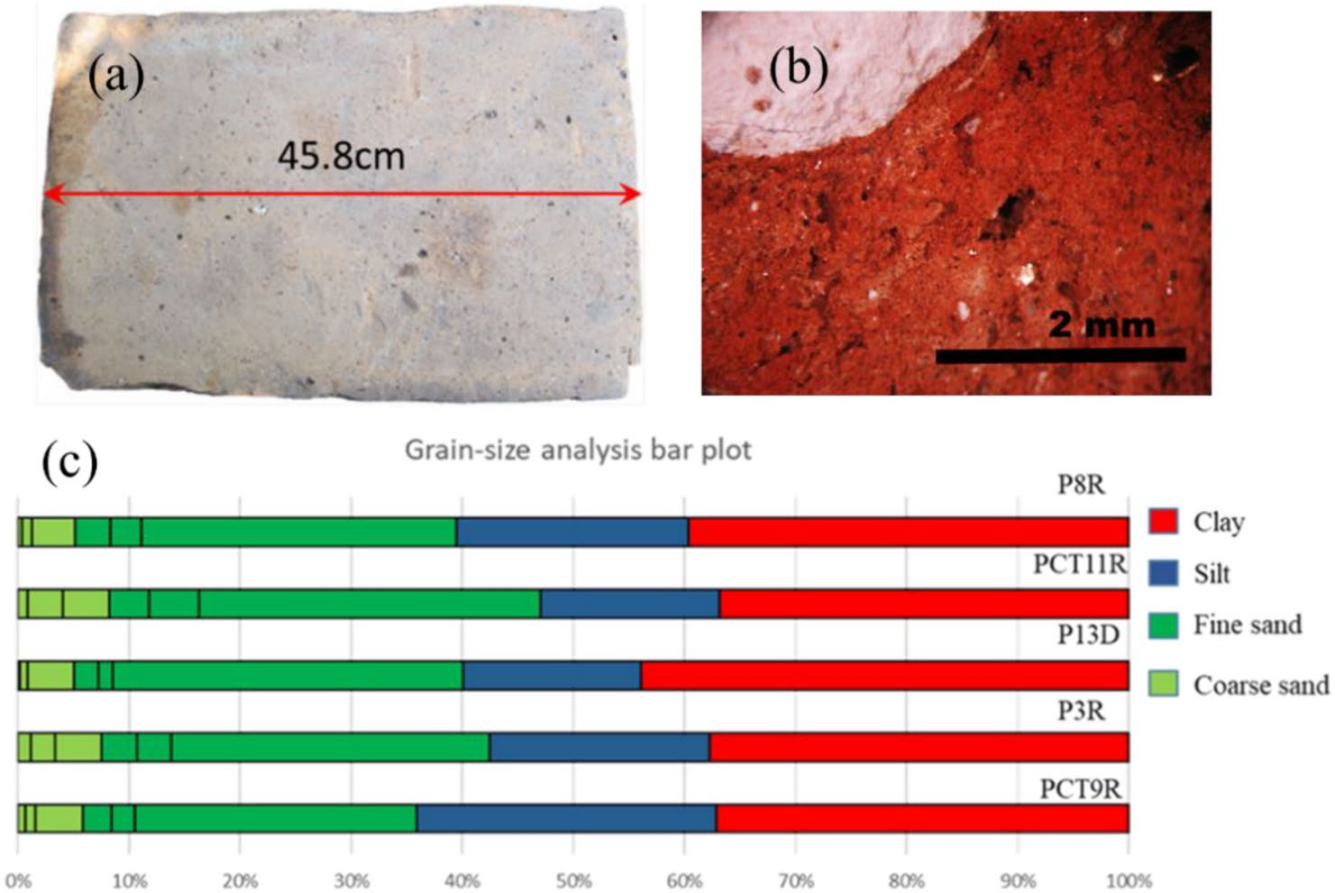
(**a**) The as-excavated burnt brick (denoted B) from Romula. (**b**) Detailed optical image showing the brick B petrographic texture (cross section): note the large white inclusion of CaCO_3_ (see text, “Discussion”). (**c**) Particle-size analysis bar plot of the small depth clay soils from Table 1. For the clay soil horizon see Fig. 1b. Fractions (from right to left) are: < 0.002 mm for clay; 0.002–0.02 mm for silt; 0.02–0.05 mm, 0.05–0.1 mm, and 0.1–0.2 for fine sand; 0.2–0.5 mm, 0.5–1 mm, and 1–2 mm for coarse sand. For sample notations see Table 1.

Excavations performed recently (2017–2018) at the Central Fortification (*castra*) revealed archaeological structures built with burnt bricks (*lateres cocti*, last decade of the second century to first third of the third century AD) and unburned (mud) bricks (*raw lateres*, first quarter of the second century AD) of *sesquipedalians* type (Fig. 1c,d). The identification of the period when the construction was made was based on the coins found in the walls. The discovery of mudbricks at Romula, i.e. to the north of the Roman Empire came as an unexpected surprise since such bricks are used in e.g. Egypt^10^ where the climate is hot and with a low level of rainfall. No report on Roman mud bricks from this colder region was found. Production and use of mud bricks at Romula can be explained by the presence of the permanent unit of Syrian archers (*numerus*)^11^. They were probably accustomed to mudbricks fabrication and use. The mudbricks sizes show a larger variation than for the burnt bricks. A typical burnt brick with the Length x Width x Height of 45.8 cm × 29.7 cm × 6.2 cm is presented in Fig. 2a,b.

### Samples physical characterization

The apparent porosity (*P*), water absorption (*Abs*) and bulk density (including pores, *ρ*, and of the material without open pores, *R*) are the first analyzed properties of burned bricks. These properties are widely used in the evaluation and comparison of the brick’s quality. The Archimedes procedure and calculations were applied according to *C134* and *C830* ASTM standards (Supplementary material Table 1). Measurements were performed with a KERN ALT220-4M density balance (± 0.00001 g error).

The burnt brick was observed with an in-lab built micro-tomograph^12,13^. More details of the method are presented in Supplementary material Table 2.

The fractions of clay, silt, fine and coarse sand from the raw clay soils were determined by using the sieving method.

A Bruker-AXS D8 ADVANCE powder diffractometer (CuK*α*1 radiation, *λ* = 1*.*5406 Å) was used to measure X-ray diffraction (XRD) patterns. Samples were either massive ceramics where a surface was flattened by cutting and polishing, or powders obtained by manual grinding with an agate mortar and pestle. Minerals were identified based on international databases of diffraction files and refs.^8,19^. Because of peaks overlapping belonging to multiple phases, the complexity of XRD patterns is high. Therefore, to identify phases by narrowing the possible mineral classes, results of XRF and FT-IR were also used. A quantitative phase analysis was attempted by Rietveld method. This analysis confirmed the presence of minerals identified for major phases (> 3–5 wt.%) and showed the possibility of the trace phases (< 3–5 wt.%) presence. Although we selected the most representative samples, we emphasize that rigorous quantitative phase analysis is challenging given the natural background of the samples^14^. Hence, our results should be viewed as qualitative. In samples PCT9R* and S were obtained the minimum (6.3%) and maximum (14.8%) values of the weighted profile R-factor (R_wp_). Results of Rietveld analysis are presented in Supplementary material Table 3.

Images of microstructure and the local maps of elements were taken by scanning electron microscopy (SEM/EDX, Zeiss EVO50). Massive samples were observed on fresh surfaces resulting from fracturing. Observation by SEM of dielectric samples as in this case is difficult. Hence, our samples were covered with a conductive layer of Au deposited by vacuum evaporation.

X-ray fluorescence spectrometry (XRF) was employed (Bruker S8 Tiger with two detectors, one proportional flow counter and one scintillation counter) to determine the chemical composition of the samples. The X-ray tube has an Rh anode of 50 kV and 50 mA with a 75 μm Be-window. The instrument is equipped with XS-55, PET, LiF (200) analyzer crystals. The measurement method (in 1 atm He) was selected in the QUANT EXPRESS software to be “Full Analysis”. The concentration values are presented as a *standard-element-oxide-conversion* table. Three measurements were performed for each sample in the powder state (grounded for homogenization with an agate mortar and pestle) and the average wt. % for an oxide was determined (Table 2). The weight percentages were normalized to 100 wt.% for the main oxides: SiO_2_, TiO_2_, Al_2_O_3_, Fe_2_O_3_, MgO, CaO, Ka_2_O, Na_2_O, P_2_O_5_, MnO, and BaO. Statistical errors provided by the XRF machine on our samples were about 0.63%, 2.53%, 1.36%, 0.519%, 3.89%, 1.15%, 1.37%, 12%, 5.81%, 4.65%, and 17.8% for the indicated oxides, respectively. Other detected elements were traces of Zr, Sr, and Zn. For the calibration, standard procedures specific for our XRF device and the reference XRF-monitor-glasses recommended by the equipment supplier were used. We also made additional checking by measuring powder mixtures of rare earth oxides with established ratios of the components. Samples were 34 mm in diameter, and 5 mm in height. Powders were lightly pressed in the standard plastic holders used with the indicated XRF equipment.

**Table 2:** X-ray fluorescence average compositions (wt.%) in the oxide representation (the total is 100 wt.%) considering the main detected elements in the burnt (B) and mud (S1-1a, S1-1b, S1–2) bricks, raw sand (S), raw soils (low- and high- depth) from Romula.

Sample	Main detected elements recalculated as simple (arbitrary taken) oxides											Trace elements			L.O.I (%)
	SiO_2_ (%)	Al_2_O_3_ (%)	Fe_2_O_3_ (%)	K_2_O (%)	TiO_2_ (%)	MgO (%)	CaO (%)	Na_2_O (%)	P_2_O_5_ (%)	MnO (%)	BaO (%)	Zr (ppm)	Sr (ppm)	Zn (ppm)	
P8R	62.3 ± 0.006	16.7 ± 0.5	10.3 ± 0.6	4.18 ± 0.19	1.64 ± 0.12	1.59 ± 0.06	2.12 ± 0.18	0.472 ± 0.2	0.155 ± 0.02	0.178 ± 0.03	0.156 ± 0.05	1120 ± 112	338 ± 22	291 ± 70	4.1
P3R	63.6 ± 0.001	16.3 ± 0.4	9.43 ± 0.4	4.73 ± 0.01	1.54 ± 0.05	1.52 ± 0.01	1.79 ± 0.08	0.596 ± 0.13	0	0.23 ± 0.03	0.126 ± 0.05	924 ± 110	343 ± 21	200 ± 19	3.8
PCT9R	62.6 ± 0.001	16.6 ± 0.2	10 ± 0.2	4.2 ± 0.09	1.64 ± 0.03	1.53 ± 0.06	2.29 ± 0.14	0.572 ± 0.07	0.177 ± 0.01	0.196 ± 0.001	0	1030 ± 300	300 ± 11	209 ± 15	4.5
PCT9Rp	62.8 ± 0.01	16.3 ± 0.1	9.94 ± 0.4	4.18 ± 0.18	1.67 ± 0.04	1.6 ± 0.12	2.41 ± 0.11	0.507 ± 0.15	0.202 ± 0.015	0.2 ± 0.02	0	1010 ± 200	360 ± 10	221 ± 5	4.5
B	61.35 ± 0.005	14.85 ± 0.15	7.035 ± 0.1	3.96 ± 0.08	1.195 ± 0.05	2.02 ± 0.02	7.85 ± 0.01	0.892 ± 0.06	0.53 ± 0.001	0.127 ± 0.005	0	790 ± 20	384 ± 16	195 ± 22	0.9
S1-1a	58.3 ± 0.001	12.1 ± 0.21	6.53 ± 0.15	3.37 ± 0.03	1.08 ± 0.1	1.47 ± 0.03	15.9 ± 0.06	0.436 ± 0.002	0.505 ± 0.001	0.0845 ± 0.003	0	585 ± 14	713 ± 23	175 ± 31	0.8
S1-1b	56.5 ± 0.002	11.5 ± 0.23	6.5 ± 0.2	3.11 ± 0.02	1.04 ± 0.05	1.44 ± 0.05	18.9 ± 0.07	0.379 ± 0.08	0.371 ± 0.02	0.137 ± 0.007	0	552 ± 17	759 ± 19	189 ± 10	0.9
S1-2	62.2 ± 0.002	13.7 ± 0.16	7.85 ± 0.1	4.32 ± 0.01	1.46 ± 0.09	1.8 ± 0.07	6.95 ± 0.13	0.534 ± 0.06	0.789 ± 0.01	0.159 ± 0.003	0.0804 ± 0.002	933 ± 11	570 ± 5	301 ± 17	1.1
DS1-2.5m	56.6 ± 0.005	14.8 ± 0.45	9.81 ± 0.5	4.03 ± 0.1	1.49 ± 0.08	1.61 ± 0.03	10.5 ± 0.1	0.467 ± 0.03	0.262 ± 0.02	0.189 ± 0.04	0.132 ± 0.06	999 ± 7	463 ± 13	280 ± 6	0.5
DS2-3m	40.4 ± 0.006	9.85 ± 0.3	7.33 ± 0.1	2.88 ± 0.07	1.13 ± 0.08	2.16 ± 0.02	35.2 ± 0.12	0.304 ± 0.1	0.252 ± 0.01	0.16 ± 0.02	0.159 ± 0.07	663 ± 23	1010 ± 100	162 ± 11	0.1
S	76 ± 0.001	11.5 ± 0.2	3.24 ± 0.4	3.35 ± 0.02	0.686 ± 0.09	1.09 ± 0.01	3.36 ± 0.1	0.497 ± 0.007	0	0.0667 ± 0.002	0	241 ± 15	256 ± 8	0	2.1

Standard deviations from the average values for three measurements on each sample are given. L.O.I values were estimated after heating the samples at 100 °C.

The Fourier-transformed infrared (FT-IR) spectra were measured with a JASCO 4200 equipment with Pike ATR unit, in the 400–4000 cm^−1^ range.

The thermal analysis experiments were undertaken with a SETARAM Setsys Evolution 18 Thermogravimeter (Al_2_O_3_ crucibles) in the DTA–TG mode in the temperature range 20–1200 °C. The atmosphere was synthetic air (20% O_2_, 80% N_2_) with a flow gas rate of 16 ml/min. The heating and cooling rates were 10 °C/min. The initial mass of the sample was 30 mg. The accuracy of heat flow measurements was ± 0.001 mW, and the temperature precision was ± 0.01 °C. In some cases, during thermal analysis experiments, a mass spectrometer (QMS 301 Omnistar Pfeiffer) was connected.

^57^Fe Mössbauer spectroscopy studies were performed with a conventional spectrometer, using a ^57^Co (Rh matrix) source, mounted on a drive unit working in the constant acceleration mode. Powder samples were introduced into a He closed cycle cryostat and spectra were acquired in transmission geometry at 300, 80 and 5 K. The NORMOS computer program^15^ has been used for the least squares fitting of the Mössbauer spectra. The isomer shifts are reported relative to α-Fe at room temperature.

Magnetization curves with magnetic field, *M*(*H*), or with temperature, *M*(*T*), were measured with a SQUID magnetometer (MPMS-7T, Quantum Design, US) on powder samples.

Flash calorimetry (Netzsch GmbH LFA 457 Microflash) from room temperature to 500 °C was applied to measure thermal diffusivity *α* (m^2^ s^−1^) and specific heat *c*_p_ (J g^-1^ K^−1^) of the disc samples (~ 9.9 mm in diameter and ~ 1.15 mm thickness) cut from the burnt brick. Thermal conductivity *k* (W m^−1^ K^−1^) was calculated based on (Eq. 1):

1 $$k = \alpha \times R \times c_{{\text{p}}} ,$$

where *R* is the bulk density of the material, without open pores (Eq. 7, Supplementary material Table 1).

Hardness HV_0.5_ of the burnt brick was evaluated on CV-400DTS Vickers hardness tester, with a dwell time of 30 s, under a load of 0.5 kgf (4.90 N). Compressive tests were performed at room temperature with Instron 5982 system. Vickers hardness was also measured on ceramic samples prepared in the laboratory from the sieved powder fraction (as-extracted raw soil PCT9R and mudbrick S1-2) less than 100 µm. After mixing with water (~ 1.55 g/ml), samples were dried in the air for 48 h on a porous alumina bed. Dried compacts were burnt in the air in an electric furnace at 800, 850 and 900 °C for 15 h. Samples were cylinders with a diameter of ~ 10 mm and a height of ~ 8 mm.

Experimental results from compressive tests were used as input data for finite element analysis (FEA) in Inventor Nastran 2022 of the brick’s mechanical behaviour. Though the brick is a heterogeneous material, a composite-like, the experimental results represent an average value: in our FEA we considered the material being homogenous and isotropic. For meshing, we used parabolic element order with a 5 mm element size. The resulting number of nodes and elements were 710,847 and 519,813, respectively.

The colorimetric analysis of the samples was performed in two ways:

(i) sample illumination with a D65 (i.e. the International standard Artificial Daylight where the color temperature is 6500 K, CRI is 98) light source PHILIPS Master TL-D 90 De Luxe 36W/965 D65. The images of the reflected light from the sample were analyzed with the Soil Analysis Pro software;
(ii) spectroradiometric analysis (Minolta CS-2000) was performed on the sample surfaces under 405 nm. The blue light is absorbed much faster than the other colors: clays and bricks samples may contain goethite as a synthetic pigment, widely used as a UV absorber^16^. The UV–Vis spectra are characteristic of absorption bands associated with the colour (400–760 nm), with an absorbance between 0.6 and 1.0 below 500 nm, depending on the overall difference in the particle size distribution^17^. Calibration was performed with a Minolta standard CSA 5 white calibration plate before each measurement.

Colors were defined with the Munsell Soil-Color Charts, and CIE L*a*b* (International Commission of Illumination, 1986) color space. We also used the CIE derived models: simplified CIE 1931 x,y coordinates (where x and y defines the chromaticity of the light) for 2D representation and the CIE XYZ (chromaticity is specified by parameters X and Y which mark the color shift from the white light). The Munsell Soil-Color Charts are considered to reasonably cover soils as well as unglazed ceramic artefacts^18^.

## Results

### Soils characterization and their identification as raw materials for fabrication of the ancient mud and burnt bricks

The investigated low-depth (< 1.5 m) soils (Fig. 2c) show a reasonably low variation of the particles’ fractions. The fraction of the smallest particles (< 0.002 mm) is ascribed to clay and represents 37–43 wt.%. The largest particles are of sand in the range of 1–2 mm. The composition of these soils is typical for bricks fabrication. In general, they should fulfill two conditions: (1)—the amount of clay is minimum ~ 20 wt.% (sometimes even lower for mudbricks, down to 6–10 wt.%^10^), and often reaches 40 wt. %, and (2)—sand fraction is composed of relatively small size particles around 1 mm. For example, the soils considered as being used for fabrication of the Roman burnt bricks/ceramics from approximately the same period in Liguria (first to second centuries AD^8^) and Alpes Cottiae (first to third centuries AD^8^) contain 20% and 40 wt. % of clay, respectively. These soils were extracted from a depth of 1.2–1.5 m. Clay particles for fabrication of Roman and Byzantine burnt bricks used to build monuments in Greece (second to fifteenth century AD) were found to be usually below 2 µm and almost hexagonal in shape^19^. The mudbricks extracted from a building (Q 13-1 Phase A) at Tell Timai (Egypt) dated from the Roman period (second century AD^10^) contains clay between 10.65 and 30.2 wt.%. Since in our case the petrographic composition of the low-depth soils does not change significantly with the depth and considering the proximity of the soil horizon to the furnaces for bricks production in the industrial Northern Quarter of Romula, it is inferred that the identified local geological clay material deposit can possibly serve as the source of the raw material for bricks fabrication.

**Figure 3:**
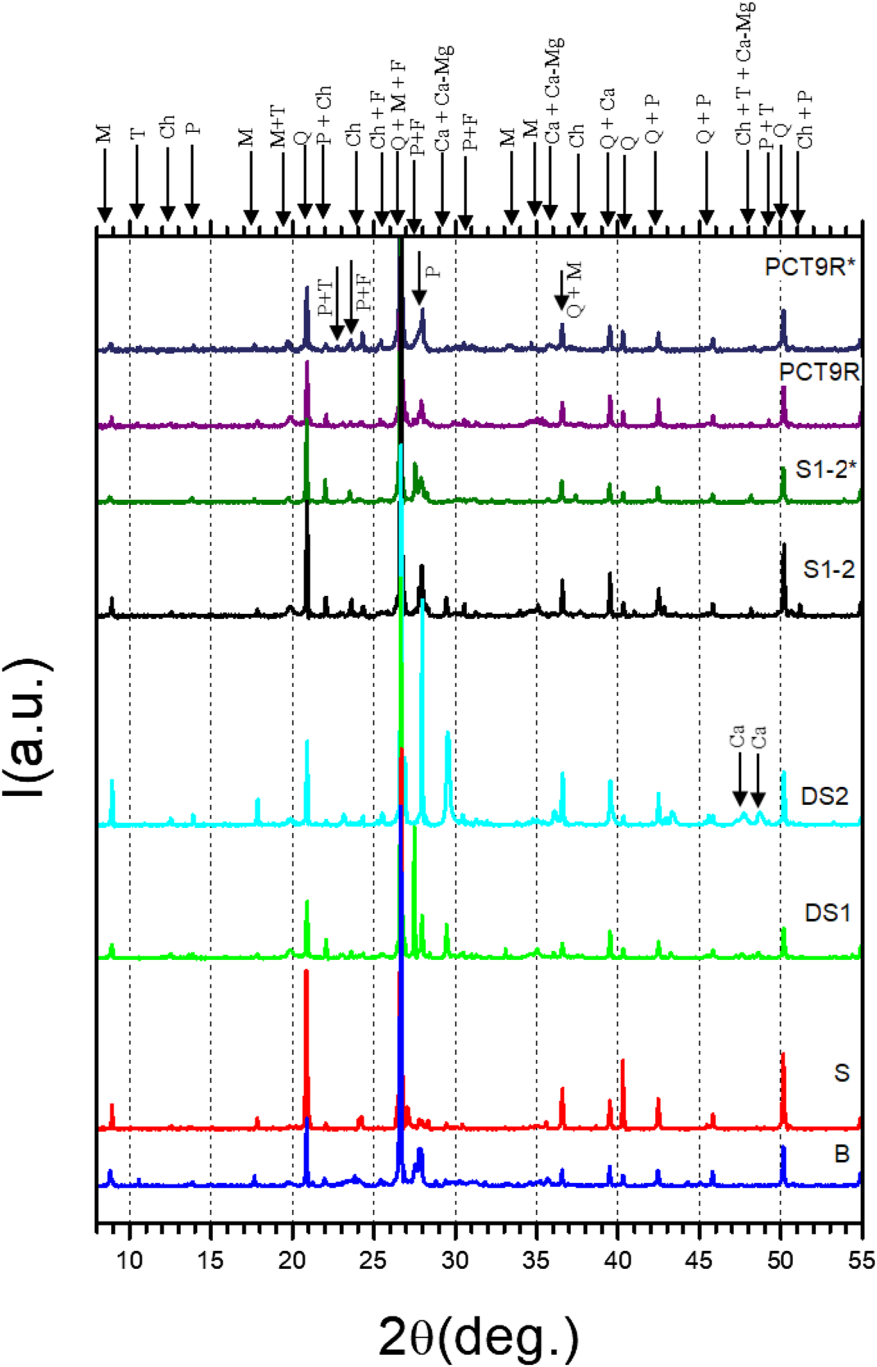
X-ray powder diffraction patterns of burnt (B) and mud (S1–2) bricks, sand (S) and raw soils (DS1, DS2, PCT9R) from Romula. The mineral phases identified are : quartz (Q, CIF: 00-033-1161), chlorite (Ch, CIF: 01-087-2496), mica (M, CIF: 01-074-1107), K-feldspar (F, 00-010-0353), plagioclase (P, CIF: 01-079-1148), Tschermakite (T, CIF: 04-012-1305), calcite magnesian (Ca–Mg carbonate, CIF: 00-043-0697) and calcite (Ca carbonate, CIF: 04-008-0198). Note: (asterisk)—powder samples ignited at 880 °C in air with a heating rate of 200 °C/h, and a dwell time of 1 h. For details of identified minerals see Supplementary material Table 4.

**Figure 4:**
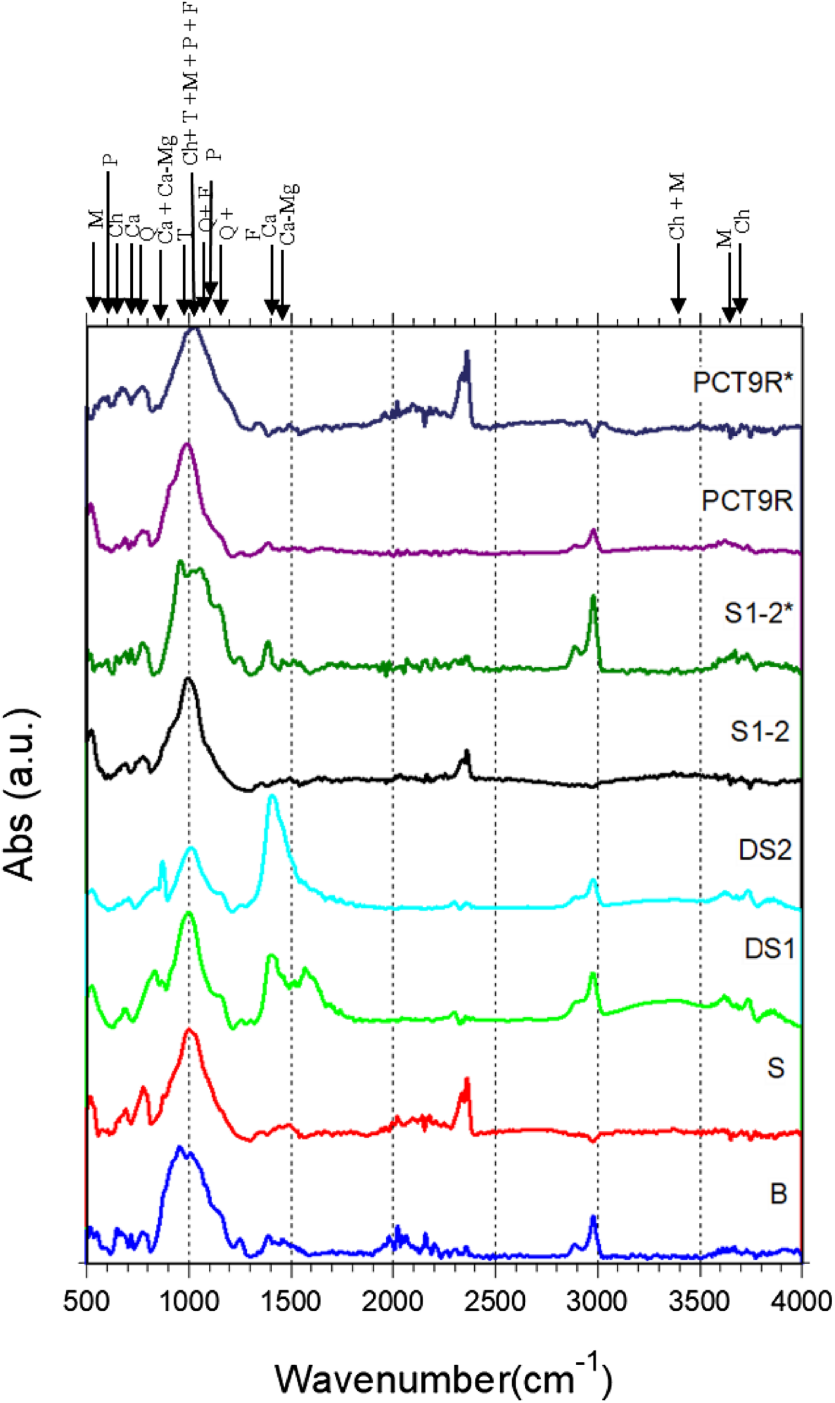
FT-IR spectra of investigated samples (see Table 1 for sample notation). Note: (asterisk) powder samples ignited at 880 °C with a heating rate 200 °C/h, and for a 1 h dwell time. The notation for the mineral phases identified is the same as in Fig. 3. For details of identified minerals see Supplementary material Table 4.

This idea is further supported by XRD (Fig. 3) and FT-IR (Fig. 4) data. Information on identified phases is summarized in Supplementary material Table 4, while results of Rietveld analysis are presented in Supplementary material Table 3. One observes that the XRD and FT-IR spectra for the mudbrick (sample S1–2) and the soil PCT9R (extracted from a relatively low depth of 1.53 m) are resembling each other. The XRD and FT-IR patterns of the other low-depth raw soils (P8R, PCT11R, P13D and P3R, Table 1) show little difference and, therefore, they are not presented. From XRD (Fig. 3, Supplementary material Table 3), in the mud brick S1–2 the amount of calcite (denoted Ca carbonate) is similar to raw soil PCT9R (under 1 wt.%). XRD (Fig. 3, Supplementary material Table 3) and FT-IR (Fig. 4) patterns for the soils DS1 and DS2 extracted from 2.5 m and 3 m (Fig. 1, Table 1), respectively, indicate the presence of a higher amount of calcite. XRD indicates 2 wt.% in DS1 and of 17.9 wt.% in DS2 of calcite (denoted Ca carbonate in Fig. 3 and in Table 3, Supplementary material). Deeper than 1.5 m, the higher the depth is, the larger amount of calcite is present in the soil. We note that calcite may contain Mg (phase denoted Ca-Mg carbonate in Fig. 3 and Supplementary material Table 3).

**Table 3:** Hyperfine parameters from Mӧssbauer spectra (Fig. 10).

Sample	Temperature (K)	Subspectra/curve notation in Fig. 10	Relative area (%)	Hyperfine field, B_hf_ (T)	Isomer shift, IS (mm/s)	Quadrupole splitting, QS (mm/s)
PCT9R	5	D_2_^2+^/D1	11	–	1.25(5)	2.81
		D_1_^3+^/C1	55	–	0.44(3)	0.65
		S_2_/B1	26	49.7(3)	0.40(5)	−0.17
		S_1_/A1	8	52.2(4)	0.40(5)	0.00
	80	DC/E2	7	–	0.20(5)	0.89
		D_2_^2+^/D2	11	–	1.33(5)	2.45
		D_1_^3+^/C2	57	–	0.38(4)	0.72
		S_2_/B2	19	47.4(3)	0.40(5)	−0.29
		S_1_/A2	6	52.1(4)	0.41(5)	0.00
	300	DC/E3	33	–	0.39(5)	1.03
		D_2_^2+^/D3	14	–	1.10(5)	2.70
		D_1_^3+^/C3	53	–	0.35(5)	0.57
S1–2	5	D_2_^2+^/I1	10	–	1.30(5)	2.93
		D_1_^3+^/H1	69	–	0.42(3)	0.51
		S_2_/G1	16	49.5(5)	0.50(5)	−0.26
		S_1_/F1	5	52.6(3)	0.40(5)	0.00
	80	D_2_^2+^/I2	10	–	1.17(6)	2.96
		D_1_^3+^/H2	72	–	0.40(4)	0.62
		S_2_/G2	14	48.5(5)	0.51(5)	−0.22
		S_1_/F2	4	51.0(3)	0.40(5)	0.10
	300	DC/J3	19	–	0.44(4)	1.07
		D_2_^2+^/I3	11	–	1.12(3)	2.7
		D_1_^3+^/H3	70	–	0.35(4)	0.6
S1–2*	5	Sing1/N1	9	–	0.12(3)	–
		D_1_^3+^/M1	19	–	0.34(5)	1.68
		S_2_/L1	35	48.1(5)	0.27(8)	−0.07
		S_1_/K1	37	52.3(5)	0.46(6)	−0.18
	80	Sing1/N2	50	–	0.26(3)	–
		D_1_^3+^/M2	5	–	0.39(5)	0.00
		S_2_/L2	19	48.0(7)	0.43(8)	−0.36
		S_1_/K2	26	52.3(7)	0.33(6)	0.07
	300	D_1_^3+^/M3	12	–	0.28(5)	0.00
		DC/N3	63	–	0.21(3)	0.86
		S_1_/K3	25	49.76(3)	0.25(6)	−0.13
B	5	D_1_^3+^/Q1	51	–	0.39(4)	1.37
		S_2_/P1	23	49.4(4)	0.32(3)	0.00
		S_1_/O1	26	53.0(5)	0.41(5)	0.00
	80	DC/R2	8	–	0.38(3)	1.83
		D_1_^3+^/Q2	52	–	0.36(3)	1.01
		S_2_/P2	21	49.6(4)	0.35(3)	0.18
		S_1_/O2	19	53.0(5)	0.40(5)	−0.1
	300	DC/R3	10	–	0.35(3)	1.8
		D_1_^3+^/Q3	48	–	0.34(3)	0.00
		S_2_/P3	22	27.0(3)	−0.13(3)	0.00
		S_1_/O3	20	48.8(5)	0.46(5)	0.00

Notations for the Mӧssbauer spectral components are *S* sextet, *D* doublet, *Sing* singlet.

XRD patterns (Fig. 3, Supplementary material Table 3) of the samples S1–2^*^ and PCT9R^*^ burnt at 880 °C in the air for 1 h and of burnt brick B are also much alike although some differences and scattering in the amount of some phases were noted (e.g., for the burnt raw soil PCT9R*, burnt mud brick S1–2* and ancient burnt brick (B), the amounts of minerals were: quartz (Q) 27.4, 34.6, and 26 wt.%; chlorite (Ch): < 1 wt.%; mica (M): 27.4, 20.2, and 19.9 wt.%; feldspar (F): 5.8, 2.8, and 8.6 wt.%; plagioclase (P): 13.6, 21.1, and 17.2 wt.%; tschermakite (T): 24.6, 20.4, and 21.8; calcite (Ca carbonate): < 1, < 1, and 1.2 wt.%; calcite with Mg (Ca–Mg carbonate): < 1, < 1, and 4.8 wt.%).

**Figure 5:**
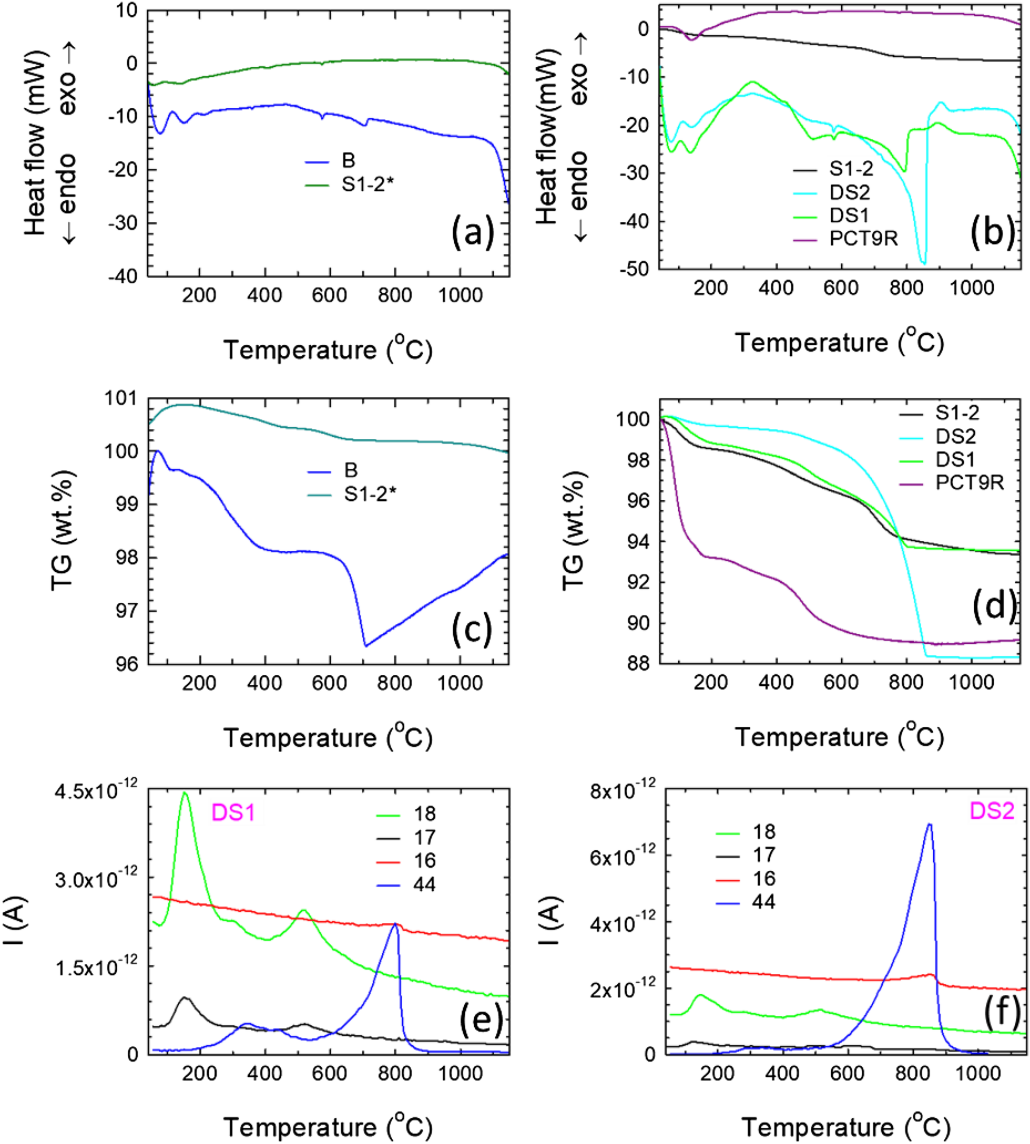
Thermal analysis and mass spectroscopy curves: (**a,c**) DTA and TG curves for samples B and S1–2^*^; (**b,d**) DTA and TG curves for PCT9R, DS1, DS2 and S1–2; (**e,f**) mass spectroscopy curves with temperature (fragments and/or molecules with mass 17, 18 and 44 were ascribed to OH, H_2_O and CO_2_) for samples DS1 and DS2, respectively. Decomposition stages in DTA/TG for each sample are given in Supplementary material, Table 5).

XRF results are presented in Table 2. Composition of the low-depth soils (0.45–1.5 m, samples P8R, P3R, PCT9R/PCT9Rp) regardless of their excavation depth varies within a relatively narrow range as anticipated from the petrographic analysis. The composition of the low-depth soils is similar to that of the burnt brick B. The only notable difference is encountered for Ca. Namely, the amount of this element is higher in the burnt brick B than in the low-depth soils. This result is supported to some degree by XRD results (compare the amount of Ca and Ca–Mg carbonate phases for samples B and PCT9R, Supplementary material Table 3). A high amount of calcium (represented as oxide in XRF data, Table 2) is found also in samples S1-1a and S1-1b extracted from the mud brick. However, some parts of the mud brick (sample S1–2) show a similar value of Ca as in the burnt brick B. On average, the mud brick exhibits a higher calcium content than in the low-depth soils and in the burnt brick B. On the other hand, a high calcium content was found in the high-depth soils DS1 and DS2. Soil DS1 extracted from a depth of 2.5 m matches well the composition of the mud brick samples S1-1a and S1-1b. One supporting information of this result is provided by thermal analysis data (Fig. 5d, Supplementary material Table 5) since the sample S1–2 from the mudbrick and the soil DS1 have a similar level of weight loss and the TG curve profiles are almost identical. Shapes resemblance of the thermal analysis curves indicate similar processes that should involve certain compounds, this suggesting that DS1 soil can be the raw material for fabrication of the mud brick. Roughly, considering also literature data, processes that occur in our samples during heating are indicated in Supplementary material Table 5. More research is needed to reveal in detail the behavior of the phases during heating.

For soils, (in XRF Table 2 and using oxide representation) a higher calcium content is accompanied by a visible decrease of Si and Al. This trend is valid also for the compositions measured on the mud brick (S1-1a, S1-1b and S1–2) and suggests in a first approximation that the investigated soils were possibly used as raw materials and in the as-extracted state without additives. An argument apparently favorable to this statement is that the use of raw materials that need as low as possible number of processing operations during brick manufacturing is economically convenient due to minimization of the manpower and fabrication time. However, other aspects should be also considered and they are discussed in “Porosity, water absorption, mechanical and thermal insulating properties of the burnt brick from Romula”.

From the viewpoint of XRD and FT-IR results, the burnt brick B from Romula shows strong similarities with the Roman tubulus (clay pipe conduct under decumanus, first to second century AD, sample IX) from Hasta, Liguria, Italy^8^. But, based on XRF the burnt bricks from Italy and Spain reported in ref.^8^ contain rare earth elements used as markers. In the local soils, mud, and burnt bricks from Romula rare earth elements were not identified. The already mentioned burnt Roman tubulus from Hasta, Liguria, Italy (sample IX, second century AD^8^) contains hematite according to XRD. Hematite was not detected in XRD and FT-IR of the burnt brick B from Romula. In the XRD pattern of the burnt brick B from Romula traces of chlorite might be present. This mineral was not found in XRD patterns of the burnt bricks investigated in ref.^8^. Burnt Roman bricks from the Serapis Temple, Bergama, Turkey (first half of the second century AD^20^) contain traces of Pt and Cu. These elements were not found in materials from Romula. Interestingly, the burnt brick from Romula has an elemental composition (XRF, oxide representation) similar to that of the big bricks from China manufactured during Ming Dynasty^21^.

Results provided in this section by XRF, XRD, FT-IR methods and thermal analysis correlate well. Considering also the context of local geography, geology, and economic life in Romula (the requirement of raw materials and water proximity to bricks fabrication area), they indicate with a relatively good level of confidence that ancient mud and burnt bricks from Romula were fabricated from the local investigated soils. Burnt bricks were probably made from low- or intermediate-depth soils (~ 1.5 to 2.5 m), while the mud bricks were fabricated from the high-depth soils (≥ 2.5 m). More details and possible implications are addressed in “Discussion”.

### Porosity, water absorption, mechanical and thermal insulating properties of the burnt brick from Romula

The burnt brick (B) has a porosity (*P*) of 39.4%, water absorption (*Abs*) of 24.9%, brick bulk density (*ρ*) of 1.57 g/cm^3^, and bulk density of the brick material (*R*) of 2.6 g/cm^3^. These values match well the reported results. In ref.^20^ for the burnt bricks from the Serapis Temple in Pergamon (today Bergama), Turkey (first half of the second century AD), the average measured values are: porosity *P* = 34.95%, water absorption *Abs* = 21.47%, bricks bulk density *ρ* = 1.63 g/cm^3^ and bulk density of the brick material *R* = 2.5 g/cm^3^.

**Figure 6:**
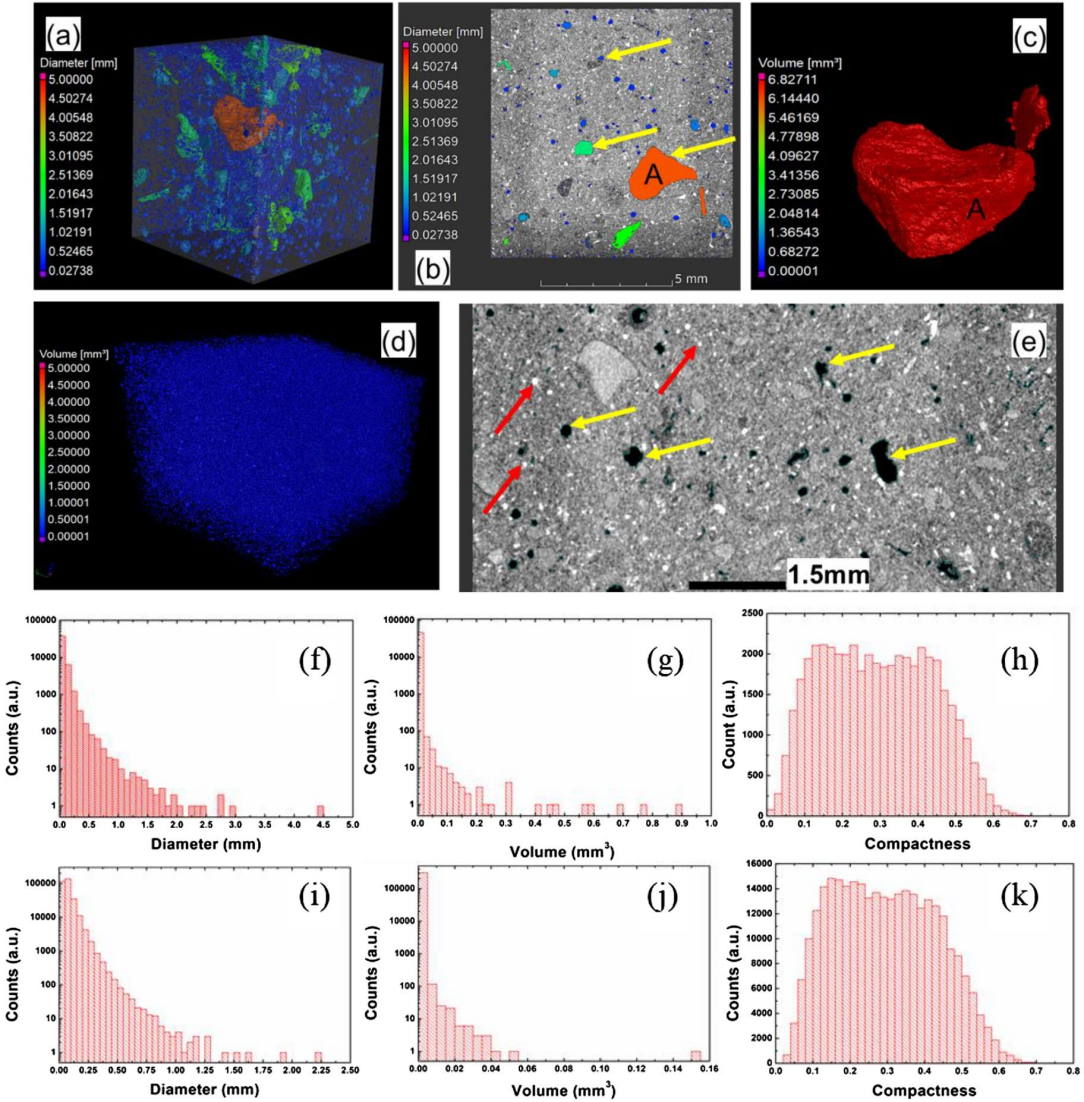
X-ray tomography on a sample from the burnt brick (B): (**a**) 3D rendering showing the pores; (**b**,**e**) 2D sections; (**c**) 3D rendering of the largest pore identified in the sample; (**d**) white particles mainly ascribed to quartz phase (see text); (**f**–**h**) diameter, volume and compactness distributions of the pores in the investigated sample; (**i**–**k**) diameter, volume and compactness distributions of the white particles in the investigated sample. Yellow arrows indicate the pores and red ones the white particles.

According to ref.^22^, large pores in the brick decrease the quality. XRT analysis of the burnt brick from Romula is presented in Fig. 6. Pores and their distribution with a size larger than 30 µm diameter are presented in Fig. 6a–c,f–h. The largest pore attains a diameter of the surrounding sphere of 4.47 mm and a volume of 0.88 mm^3^. The maximum, relatively constant compactness is in the range of 0.15–0.45. Compactness shows deviation from the circumscribed sphere around the pore where a higher compactness means that the shape is closer to a sphere. It is calculated with the formula:

2 $${\text{Compactness }} = {\text{ Volume}}_{{\text{of the pore}}} /{\text{ Volume}}_{{\text{of circumscribed sphere}}}$$

The particles of intense white color (> 30 µm) indicate on their high radiological density. The theoretical densities of the minerals identified by XRD in our samples are not much different (Supplementary material Table 4). Hence, it is impossible to distinguish them among the other phases by XRT. From the microscopy analysis (see “Microstructural aspects of the burnt brick from Romula”, Fig. 12), the largest, relatively well distributed are the particles of silicon oxide. Therefore, we shall consider that most of the as-revealed large white particles in XRT are of quartz (SiO_2_), while smaller ones may also belong to other phases. The largest white particle from the investigated burnt brick sample has a diameter of 2.22 mm, a volume of 0.53 mm^3^, and the compactness is relatively constant or it has a decreasing trend between 0.15 and 0.45.

Relatively low compactness values suggest that many large pores and the white particles (ascribed mainly to quartz) have an irregular shape as it can be directly visualized in Fig. 6a–e. The volume occupied by the large pores is 29.88 mm^3^ from the total analyzed material-volume of 821.91 mm^3^ corresponding to a porosity of 3.51 vol.%. Notably, the percentage of large pores is low. For the white particles, the calculated volume ratio is 3.14 vol.%, i.e. it is also low (the total volume of the analyzed material is 824.66 mm^3^ and the volume of detected quartz particles is 26.70 mm^3^).

**Figure 7:**
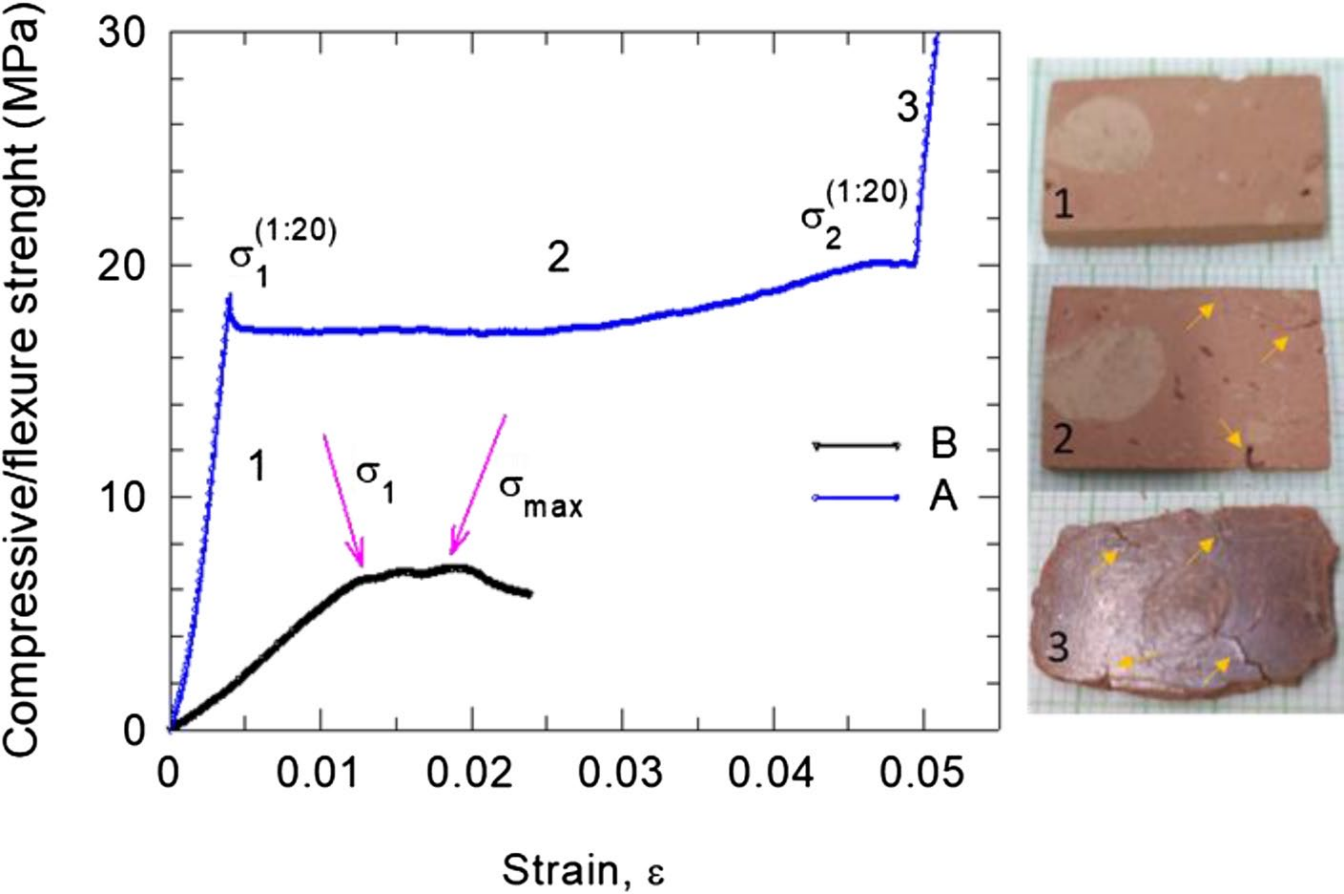
Compressive strength curves taken on the burnt brick from Romula: (**A**) compressive curve of the samples at a scale size 1:20 of the original burnt brick; (**B)** compressive curve on cubic samples (standard conditions EN1926:2006). Notations are: σ_1_—compressive strength at the first crack (curve B); σ_max_—maximum compressive strength (curve B); σ_1_^1:20^—compressive strength at the first crack (curve A); σ_2_^1:20^—intermediate compressive strength (curve A); region 1—0–σ_1_^1:20^ first crack formation (curve A); region 2—σ_1_^1:20^–σ_2_^1:20^ cracks development and compaction of the sample (curve A); region 3—> σ_2_^1:20^ compact material almost without pores.

Compressive tests on cubes with a side size of 20 and 30 mm or cylinders with a diameter of 30 mm and height of 35 mm were performed. Samples with pores less than 2 mm were selected. The tests were conformed with standards^23^. A typical curve is presented in Fig. 7. The average value of the strength at the first crack *σ*_1_ is 5.4 MPa and the maximum strength *σ*_max_ is 8.5 MPa. The Young modulus of elasticity, *E*_compressive_, was taken as the slope of the linear part of the strength-strain curve in the range of 20–60% of *σ*_1_ and has an average value of 472 MPa. The average values of *σ*_1_ and *E*_compressive_ match those measured on the bricks from Serapis Temple of 5.5/6.1 MPa and 464/423 MPa^20^, respectively. Roman bricks (second to fourth century AD) from Greece reported in ref.^19^ had a compressive strength of 4.6–18.7 MPa. Analysis and discussion regarding the mechanical behavior of the burnt brick from Romula are presented in “Discussion”.

**Figure 8:**
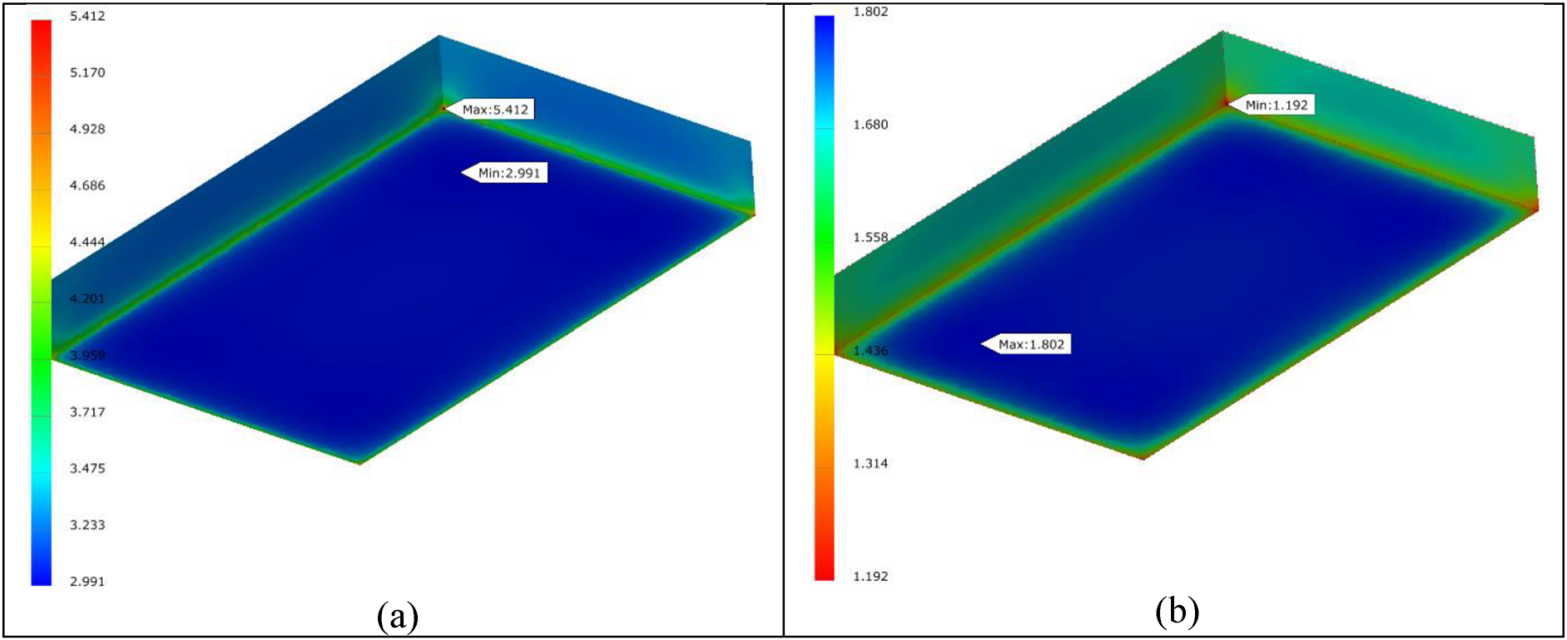
(**a**) Von Mises distribution in the first brick (from the bottom of the stack), loaded with 455 kN (about 3500 bricks). (**b**) Safety factor distribution in the first brick.

By using our experimental data, namely *ρ* = 1.57 g/cm^3^, *σ*_1_ = 5.4 MPa taken as the compressive limit, *E*_compressive_ = 472 MPa, volume and weight for one brick of 8433.612 cm^3^ and 13.241 kg, respectively, we simulated the maximum number of bricks that can be stacked in a single column to be 3500. Poisson’s ratio is ν = 0.075^24^. For a pile of 3500 bricks and based on von Mises stress failure theory we obtained a minimum safety factor of 1.192, very close to the limit (Fig. 8b). The highest von Mises stress was 5.412 MPa (Fig. 8a), and the maximum displacement was 0.469 mm. For convergence settings we used local refinement type and 1% stop criteria.

**Figure 9:**
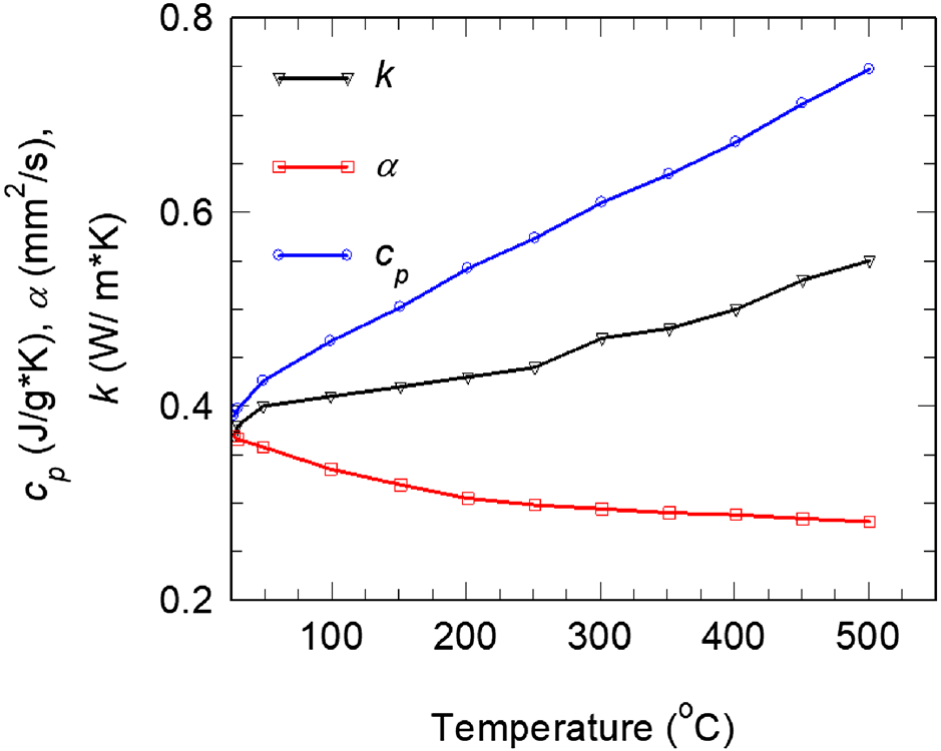
Thermal properties (specific heat *c*_p_, thermal diffusivity *α*, and thermal conductivity *k*) measured in air up to 500 °C on a sample cut from the burnt brick (B) from Romula.

Thermal properties of the ancient burnt brick material are presented in Fig. 9. The material considered without pores (*R* = 2.6 g/cm^3^) has a thermal conductivity *k* at room temperature of 0.38 W/m K (or 0.23 W/m K if considering pores, i.e. *ρ* = 1.57 g/cm^3^). It behaves like a good quality thermal insulator. For example, a burnt brick with bulk density of 1.9 g/cm^3^ considered today for the building envelope wall in tropical weather conditions had a thermal conductivity of 0.73 W/m K (specific heat *c*_p_ = 0.837 J/g K, thermal diffusivity *α* = 0.452 mm^2^/s)^25^.

### Local structure and magnetic properties of materials from Romula

It is well known that tiny differences in the macroscopic properties of the materials, and especially of composites, are related to specific local order and atomic configurations, which in some cases impose specific local interactions and magnetic properties. Therefore, the powerful technique of Mӧssbauer spectroscopy has been already used for decades to investigate local interactions and atomistic configurations including clustering processes in mineralogy^26^ as well as in studying fired clays and pottery and archeological soils^27–29^. Being an element sensitive technique, ^57^Fe Mӧssbauer spectroscopy is able to provide unmatched information about the local atomic configurations and magnetic interactions around Fe ions, even in case of tiny amounts of Fe (a few mgs) in the overall sample (100 mgs). To each Fe configuration (local arrangement of neighboring atoms/ions around the central Fe atom/ion) corresponds a spectral component and a set of hyperfine parameters (isomer shift, quadrupole splitting and hyperfine field) which define uniquely the local structure. A crystallographic phase will be therefore precisely determined by the hyperfine parameters of the corresponding local configurations (one or more), and this works also in cases of a much lower structural coherence lengths that are typically detected in XRD. In addition, the local spin configuration of each Fe position can be determined precisely by Mossbauer spectroscopy and finally the overall magnetic configuration corresponding to a crystallographic phase is revealed. The magnetic relaxation which can be nicely studied on each Fe configuration by Mössbauer spectroscopy under a very low time window of about 10^–8^ s, gives useful information on the clustering process of the Fe ions, especially when this information is corroborated with zero field cooled–field cooled (ZFC–FC) investigations. More details on the assignment of the spectral components to different Fe configurations and phases, as well as on the investigation of the clustering process via temperature dependent Mossbauer spectroscopy in connection to ZFC–FC magnetization measurements, are provided in ref.^30^.

**Figure 10:**
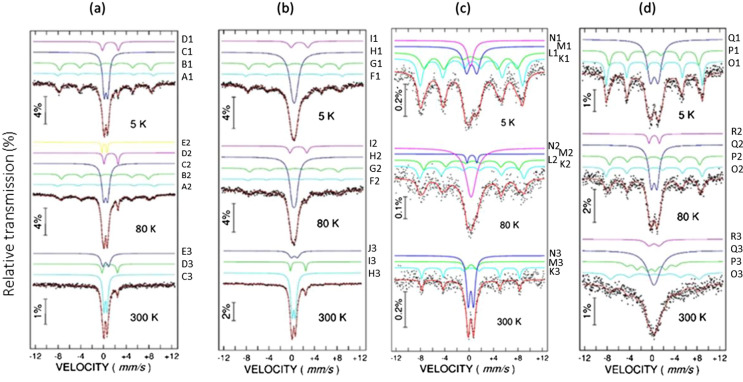
Mӧssbauer spectra at 5, 80 and 300 K measured on samples: (**a**) PCRT9R; (**b**) S1–2; (**c**) S1–2*; (**d**) B (for sample notation see Table 1). With points are experimental data.

The Mӧssbauer spectra collected at different temperatures on four representative samples (PCT9R, S1–2, S1–2*, B) show distinct features, as presented in Fig. 10.

At low temperatures (5 and 80 K), all spectra consist of a broad six-line absorption pattern and a central contribution provided by a singlet/doublet or their combination. The broad six-line pattern has been fitted by two sextets with hyperfine parameters and relative contributions being presented in Table 3. It can be observed that in the case of PCT9R (soil) and S1-2 (mud brick) the less intense outer sextet (S1 by A1 and F1 curves from Fig. 10) is characterized by a hyperfine magnetic field of about 52.5(5) T, whereas the inner sextet (S2 by B1 and G1) is characterized by a hyperfine magnetic field of about 49.5(5) T. By increasing the temperature up to 80 K, the average hyperfine magnetic field of the two sextets decreases slowly for both samples, e.g. from 50.16 T at 5 K down to 48.52 T at 80 K in PCT9R and from 50.12 T at 5 K down to 49.05 T at 80 K in S1–2. It is worth noting that the isomer shift values assigned to both sextets in the two samples (0.40(5) mm/s and 0.40(5) mm/s in PCT9R and 0.50(5) mm/s and 0.40(5) mm/s in S1–2 at 5 K) are specific to Fe^3+^ ion configurations. With a further increase of the temperature above 80 K, a part of the sextet pattern collapses with the formation of an additional contribution at the central pattern (DC by E2), thus supporting the assignment of the low temperature sextets to Fe^3+^ ions forming fine clusters of broad size distribution, with the finest ones presenting a superparamagnetic behavior above 80 K. In these circumstances, except for possible distortions induced by the atomic surrounding of the Fe^3+^ ions, a slowly decreased hyperfine magnetic field due to starting coherent magnetic relaxation phenomena might be expected for the Fe positions in the cluster as compared to the bulk-like positions. Hence, the sextet with the hyperfine magnetic field of 52.5(5) T observed at 5 K can be assigned to Fe^3+^ ions in clusters of defected hematite, whereas the one with the hyperfine magnetic field of 49.5 T to Fe^3+^ ions clusters of defected maghemite (typical values for bulk hematite and maghemite are ~ 2.0 T higher^31^). We note that the hyperfine magnetic field of 49.5 T at 5 K could also correspond to defected magnetite. While in fact maghemite itself can be seen as a cation deficient magnetite, the more general assignment of defected maghemite (maghemite + magnetite) will be considered for this less split sextet. The reason that such typical Fe-oxide phases are not evidenced via XRD (see Fig. 3) is related to their specific low structural coherence length and it is also due to many structural defects and/or small cluster size. As subsequently discussed, the characteristics of the Fe oxides in these samples are intimately related to their origin or to processing steps: using the hyperfine structure one can identify raw materials, compare artefacts and to establish their provenance, or follow and understand physical–chemical processes during processing. Therefore, ^57^Fe Mӧssbauer spectroscopy should be considered an essential powerful investigation technique that provides additional and very useful information.

For the central pattern, two doublets were considered. Their relative contributions are presented in Table 3. At 5 K, it can be seen that in the case of PCT9R and S1-2 the outer doublet (D2^2+^ by D1 and I1) is characterized by an average isomer shift value of about 1.27 mm/s corresponding to paramagnetic Fe^2+^ configurations, whereas the inner doublet (D1^3+^ by C1 and H1) is characterized by an average isomer shift value of about 0.42 mm/s corresponding to paramagnetic Fe^3+^ configurations. By increasing the temperature up to 300 K, the central patterns due to the Fe^2+^ and Fe^3+^ ions (D_1_^3+^ by C3 and H3 and D_1_^2+^ by D3 and I3) have almost the same relative contributions as at 5 K, suggesting that the corresponding ions belong to paramagnetic phases down to the lowest temperature of our measurement. To note that a part of these paramagnetic ions may contribute to Ferri-Tschermakite, as one of the better crystallized phases, well observed by XRD. However, in spite of the phase denomination related to mainly structural aspects, the random occupation of the mineral structure with various cations may lead to the presence of both species (Fe^2+^ and Fe^3+^) in this compound^32^. The rest of the Fe ions which contribute to these central Mӧssbauer patterns can enter into any other mineral phase, being dispersed at atomic level and leading to only a specific paramagnetic signal. Even Mӧssbauer spectroscopy as the most powerful method for providing local information about the Fe configurations is not able to give reliable quantitative information on the relative content of paramagnetic Fe entering each of these mineralogical phases. In total, 70–80% of the Fe ions are paramagnetic in the unburnt materials (raw soil and mud brick). By comparison with the unburnt materials (PCT9R and S1–2), the burnt bricks (S1–2* and B) present a higher relative contribution of the two sextets at 5 K (S1 by K1 and O1 and S2 by L1 and P1), showing an increased clustering by taking over of the dispersed iron. Hence, the outer sextet (S1 by K1 and O1) with the hyperfine magnetic field of 52.5(5) T observed at 5 K for both burnt bricks can be assigned to Fe^3+^ ion configurations in clusters of defected hematite. The inner sextet (S2 by L1 and P1), corresponding to a hyperfine magnetic field of 48.1(5) T for sample S1–2* and 49.4(4) T for sample B, is associated with a more defected maghemite. By increasing the temperature up to room temperature, the average hyperfine magnetic field in S1–2* is slightly decreasing and a fraction of the outer sextet collapses (S1 by K3), while the inner sextet (from L1 and L2) collapses completely. Based on changes induced by the increased temperature in the Mӧssbauer spectra of the sample S1–2*, one may assume a wide size distribution of the clusters (bimodal shape), the smaller ones collapsing, while the biggest ones, due to mainly hematite, remaining magnetically frozen even at 300 K. On the other hand, the outer sextet (S1 by O3) in sample B which is partially collapsing by increasing the temperature up to 300 K, is due to nanoparticles of hematite with a lower average size as compared to sample S1–2*. Oppositely, the inner sextet (S2 by P3) assigned to maghemite nanoparticles, presents a reduced collapsing behavior at 300 K as compared to S1–2*, thus inferring the presence of nanoparticles with a larger average size in sample B. Considering the central pattern features, namely that the outer doublet corresponding to the Fe^2+^ phase is missing, and the only paramagnetic component is assigned to dispersed Fe^3+^, the oxidation reaction induced by the thermal treatment for both bricks (S1–2*, B) is validated.

**Figure 11:**
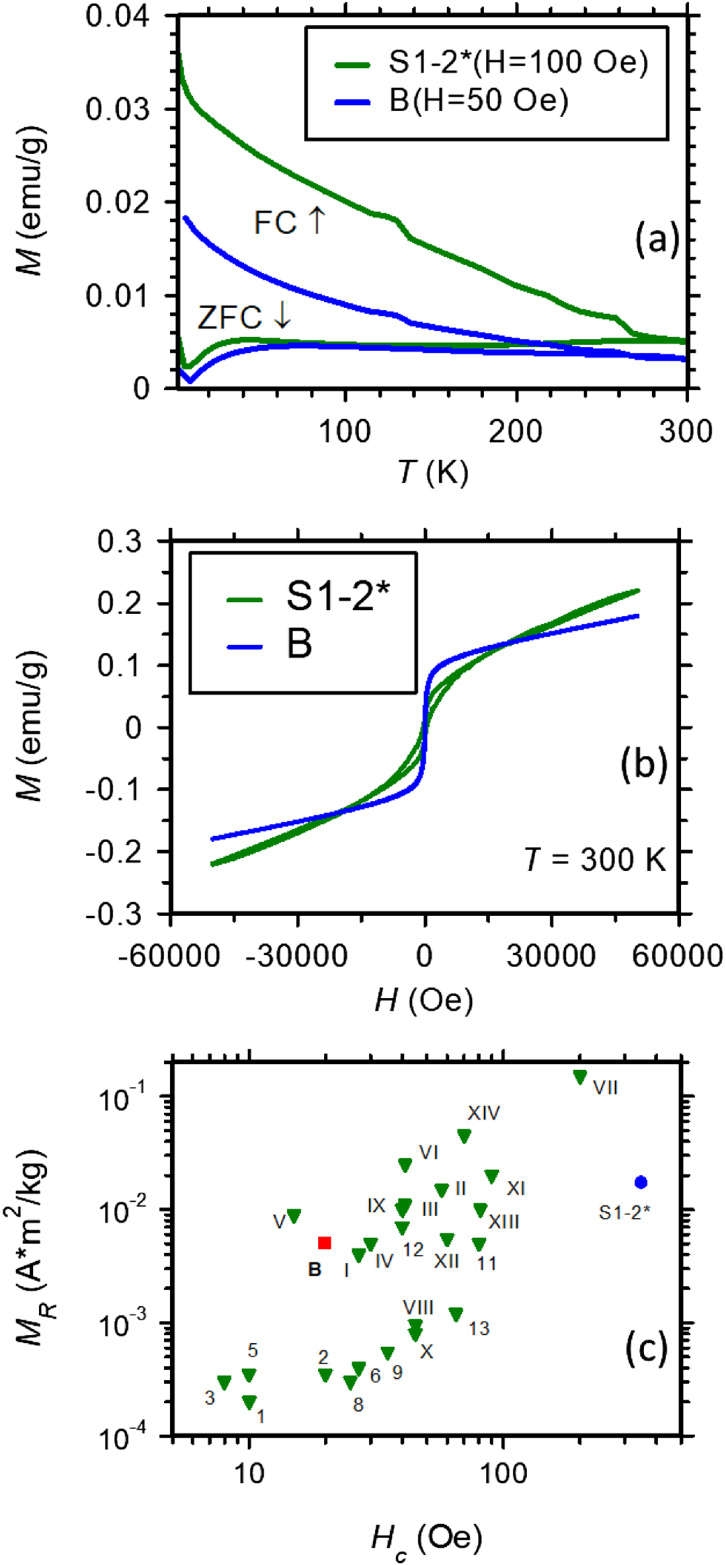
(**a**) Magnetization curves with temperature at a magnetic field of 100 Oe for S1–2* and 50 Oe for sample B; (**b**) the magnetic hysteresis curves at 300 K; (**c**) distribution of remnant magnetization (*M*_R_) against the coercive field (*H*_C_) of the burnt brick samples S1–2* and B from Romula in comparison with data for soils (Arabic numeral) and burnt bricks (Roman numeral) from ref.^8^.

The conclusion of the Mӧssbauer spectroscopy investigation is that the thermal treatment induces, on one side, an increased clusterization process of Fe and, on the other side, an increased oxidation, acting especially on the dispersed Fe ions. From this viewpoint, the Mӧssbauer results agree with the ZFC–FC data which show the following distinct features (Fig. 11a):

(i) a faster increase in both magnetization curves (ZFC and FC) at decreasing temperatures lower than ~ 5 to 10 K, specific to a transition to a ferromagnetic coupling of the dispersed Fe ions at very low temperature.
(ii) the increasing trend of the ZFC curve versus temperature with a very broad maximum between 50 and 250 K and the divergence of the ZFC and FC curves below 260 K indicate the presence of very fine magnetic clusters with a very broad size distribution, part of them being in the superparamagnetic state below 300 K. Note that such specific features do not depend on the cooling/measuring field, once it is enough weak (50 or 100 Oe in the present study) in order to introduce only a small asymmetry of the minimum energy specific to the uniaxial anisotropy of the magnetic clusters.
(iii) the specific jump in the FC magnetization curve observed at about 260 K corresponds to the spin-flop Morin type transition of hematite, giving an additional support for the presence of hematite clusters in the samples.
(iv) the specific jump in the FC magnetization curve observed at about 120 K corresponds to the Verwey transition of magnetite. However, the magnitude of the transition is very low, suggesting that only a tiny amount of enough well-structured magnetite phase can support such a transition.
(v) transitions (iii) and (iv) are better defined for S1–2* (burnt mud brick) as an indication of a narrower size distribution of magnetic clusters of hematite and defect magnetite/maghemite with a ferrimagnetic order^33^.

The above-mentioned magnetic measurements are all consistent with a superposition of magnetically ordered (cluster level, according to feature (ii)) and paramagnetic phases. None of the better crystallized silicate and carbonate phases are magnetically ordered and, therefore, the clustering process can be discussed only in relation to the Fe ions. Although the paramagnetic Fe ions are in a relatively higher amount, magnetic components with more than 20% relative spectral area can be inferred by Mӧssbauer spectra of the analyzed samples. Expectedly, if all the paramagnetic Fe ions would belong to Tschermakite (20–27 wt.%), a minimum of 3–4 wt.% of the sample would be attributed to the Fe oxide clusters which are magnetic at low temperatures. Due to their small size (nm order) and high structural disorder (both demonstrated by Mӧssbauer spectroscopy) such clusters cannot be evidenced by XRD. Their presence is however directly connected with magnetization reversal features, as remnant magnetization (*M*_R_) and coercive field (*H*_C_), which will be further discussed in the next paragraph.

The presence of the paramagnetic signal due to Fe-containing phases (Fig. 11b) can be also seen from the shape of the *M*(*H*) curve for the burnt bricks (S1–2* and B). However, the signal is not saturated as for typical paramagnetic materials. This may indicate the presence of two paramagnetic phases. The behavior of *M*(*H*) considering the remnant magnetization (*M*_R_) versus the coercive field (*H*_C_) at room temperature for the burnt bricks from Romula (S1–2* and B) is approximately similar to that of other sesquipedalian Roman burnt bricks/tiles/tubulus (from first to third centuries AD) reported in ref.^8^ (Fig. 11c). One observes that for the burnt bricks from Romula (S1–2* and B), *M*_R_ has a low variation and takes a median value among all considered soils and bricks, while *H*_C_ shows a large variation and the value for S1–2^*^ is the highest among all compared materials. A large *H*_C_ is explained by slow relaxation (lower slope) towards a steady-state in S1–2* because of many and relatively large unrelaxed particles of defected hematite also evidenced in Mӧssbauer spectra analysis. It is noteworthy that bricks from ref.^8^ contain rare earth elements that may contribute the magnetization loops whereas in the Roman brick from Romula coexist different magnetic phases. Therefore, comparative analysis of *H*_*C*_ and *M*_*R*_ of different samples is used only as indicative and without a well-established physical background.

### Colorimetric analysis of the soils and bricks from Romula

The reflection spectra measured at D65 illuminations at locations denoted L and R of the bulk burnt brick from Romula (sample B), on a flat surface obtained by grinding with sandpapers (up to a grit number of 2000), show differences (Table 4). Saturation changes from yellow 10.0 YR 5/4 in B(L) to red 7.5 YR 5/4 in B(R). This indicates that sample B presents non-uniformities. Soil constituents that influence spectral behavior are: organic matter, iron oxides and other forms of iron minerals, calcium carbonate content, mineralogy, clay, and moisture content^34^. Other factors influencing results the color measurements are roughness, particle size, powder or aggregated state of the sample^34^.

**Table 4:** Munsell colorimetry obtained as reflections of the day light equivalent illumination (D 65) compared with blue light reflections.

Sample	Munsell	CIE-L*a*b*	CIE XYZ	CIE xy (D 65)	CIE xy (405 nm)
B(L)	10.0 YR 5/4	53.92; 9.69; 20.55	22.88; 21.9; 13.61	0.3918; 0.3750	0.4506; 0.5079
B(R)	7.5 YR 5/4	49.95; 14.28; 23.28	20.24; 18.39; 10.07	0.4156; 0.3776	0.4822; 0.4863
S1–2	10 YR 8/2	79.71; -0.64; 13.80	53.14; 56.18; 47.07	0.3398; 0.3595	0.4127; 0.5335
S1–2*	7.5 YR 7/8	72.52; 15.22; 40.53	47.50; 44.44; 19.17	0.4275; 0.3999	0.4928; 0.4759
PCT9R	10.0YR 7/4	73.45; 5.34; 18.46	45.42; 45.85; 34.06	0.3624; 0.3658	0.4652; 0.4925
PCT9R*	7.5 YR 7/10	69.63; 21.99; 53.57	45.48; 40.23; 11.33	0.4686; 0.4145	0.5261; 0.4514
S1-2/850 °C	5.0 YR 4/6	41.48; 19.04; 29.03	14.23; 11.97; 4.58	0.4664; 0.3947	–
S1-2/800 °C	10 YR 4/4	40.9; 10.83; 24.28	12.77; 11.80; 5.48	0.4202; 0.4056	–

**Figure 12:**
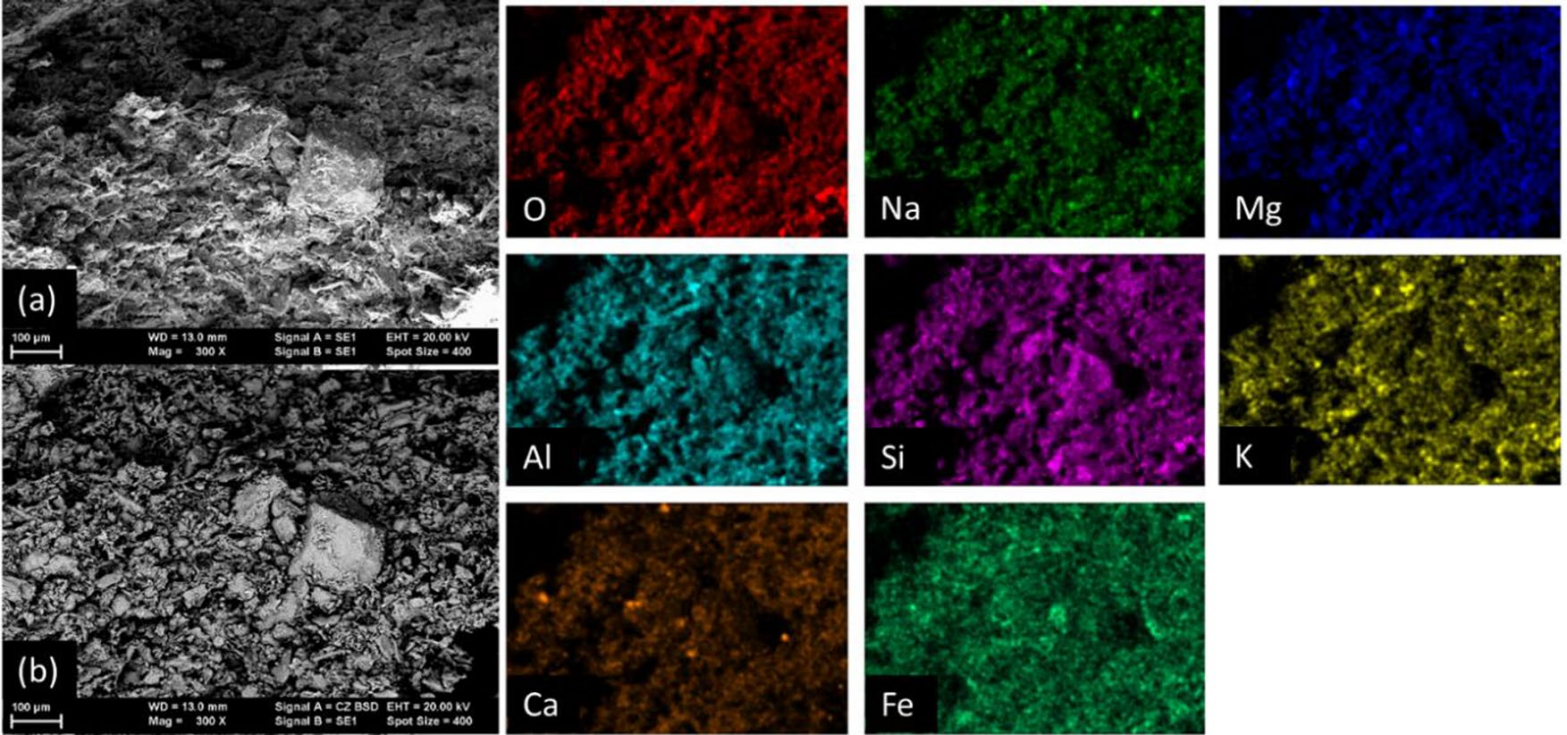
Electron microscopy images of the burnt brick B in secondary electron (**a**) and backscattered mode (**b**) and EDS maps of selected elements.

Colors of iron oxides are yellow, orange, red, brown, and brown-black. Generally, a stronger reddish pigmentation of raw and air-burnt soils is associated with the presence of hematite (α-Fe_2_O_3_). However, hematite with different colors (gray, yellow, red, violet) can be obtained depending on the precursors and calcination temperature in the air^35^. Gunal et al. by investigating different soils (CIE L*a*b* color space) concluded that + b* (b* = yellowness hue axis with negative values for blue and positive ones for yellow) could be considered a good indicator of iron oxides in any color, and + a* (a* = redness hue axis with negative values for green and positive ones for red) is a good indicator of iron oxides with a red color. Authors of ref.^34^ found that calcium carbonate enhances soil lightness L* (L* = 0 and L* = 100 correspond to no reflection of black and perfect reflection for white, respectively). When applying these results to data from ref.^35^ on pure hematite red powders synthesized at 600 °C, a* and b* values are positive and relatively high, as expected a* = 35.4–38.7, b* = 38.6–42.4, while L* = 40.6–43.5, i.e. L* takes intermediate values (average particle size 375–475 nm by SEM). For the pure hematite powders synthesized at 300 °C departing from red to orange (dull orange or light brownish) the parameters are a* = 5.6–8.5, b* = 12.1–18.4, and L* = 36.4–51.8 (average particle size 1500–4260 nm). When annealing is performed at 1000 °C, powders are bluish-gray or purplish-gray and parameters are a* = 5.2–8.2, b* = 8.6–13.5, and L* = 33.1–33.7 (average particle size 336–372 nm). It results that powders produced at 300 and 1000 °C have positive, but low b*-values, a result that does not fully support the conclusion on b* behavior of Gunal et al.^34^. Refinement in the analysis methodology is needed and we propose the following:

An interesting observation is that the ratios a*/b* < 1 and a*/b* > 1 for hematite powders synthesized at 300 and 1000 °C, respectively. Additionally, the highest value of L* was recorded for the first type of powders. A value a*/b* around 1 seems to characterize red hematite obtained at 600 °C. The proposed analysis model based on the a*/b* ratio is further applied to our materials, but before doing that, it is necessary to introduce a few key aspects that one has to take into account:

(i) The mentioned hematite powders from ref.^36^, as suggested by the authors, may contain S and C. In our materials, sulfur was not detected.
(ii) On the other hand, brick materials S1–2 and B from Romula can be classified as calcareous since they contain > 5 wt.% of CaO (XRF data in Table 2) and according to Maniatis et al.^35^ in Ca-rich clays growth of the hematite in the air at temperatures above 700 °C is suppressed. A lower amount and particle size of hematite in calcareous clays promotes a lighter color, i.e. orange instead of red in the non-calcareous clays, for firing temperatures of 700–900 °C. This effect was ascribed to ’trapping’ by the dilution of iron in the aluminosilicates: calcium aluminosilicates are stabilized by iron, for example in our case tschermakite and plagioclase that were detected by XRD and FT-IR (Figs. 3, 4) in the raw (S1–2, PCT9R) and burnt (S1–2*, PCT9R* and B) studied materials. According to ref.^35^, the consequence of Fe and Ca interplay is that stable and low-level vitrification occurs at ~ 850 °C and it does not increase with a higher temperature as in the Ca-poor clays.
(iii) The fine structure of materials from Romula (see “Local structure and magnetic properties of materials from Romula”) have shown that in the burnt ones there are different hematite types, defected or not, and with different particle sizes, but relatively small and randomly dispersed, thus, being undetectable by XRD or FT-IR (Figs. 3, 4). The SEM/EDS images taken at a magnification of 300 × on the burnt brick from Romula (B) (Fig. 12) show Fe and its non-uniform distribution (intense spots in the elemental maps indicate a higher local concentration).

If the non-uniformity of Fe distribution in the burnt brick and the presented Fe states, especially of hematite, are not considered, and given the above-mentioned details (ii), the burnt materials (S1-2* and B) should have an orange (YR) hue specific for Ca-rich clays. Indeed, there is a good agreement with the experimental results (Table 4, Supplementary material Fig. 1a). The values of a* and b* of the burnt materials S1–2* and B are 9.69–15.22 and 20.55–40.53. The value of a* is between the values for orange hematite (300 °C) and red hematite (600 °C), and the value of b* is in the range for red hematite (600 °C). The ratio a*/b* < 1, i.e. it is closer to typical values for orange hematite (300 °C). Additionaly, considering Mӧssbauer results from “Local structure and magnetic properties of materials from Romula”, the burnt soil PCT9R* thermally treated (at 880 °C, in the air) in the same conditions as S1–2* has a higher amount and larger hematite particles, as a consequence of the information provided at (ii) of the previous paragraph, i.e., PCT9R contains a lower amount of calcium (oxide representation in XRF, Table 2), 2.29 wt.%, versus 6.95 wt.% in S1–2 (Table 2). In XRD (Supplementary material Table 3) the amount of calcium-containing phases (Ca carbonate or Ca–Mg carbonate) is maybe also lower in PCT9R than in S1–2. The hematite in PCT9R* is of the red type with a* = 21.99 higher than 15.22 in S1–2*. At the same time, as expected, a*/b* is closer to 1 (0.41) in PCT9R* than in S1–2* (0.375).

The performed analysis also works well when applied to compare raw and air-burnt samples (Table 4), i.e. S1–2 and S1–2*, or PCT9R and PCT9R*. Parameters a* and b* are positive and higher in the burnt samples than in the raw powders, and L* decreases for the burnt ones.

The tubular sample IX (second century AD) from ref.^8^ with the highest resemblance in the XRD and FT-IR spectra to those of the burnt brick B from Romula has a detectable amount of hematite in XRD and its Munsell color parameters are 2.5YR 5/4 classified as dull reddish brown. The Roman bricks investigated in ref.^8^ were defined as light red and reddish brown.

Because the brick surfaces better absorb the blue light^17^, experiments were also performed for blue light illumination. Here, changes are mainly related to chromaticity shifts instead of the color saturation level. Some differences for D65 and 405 nm illumination trends were observed and they are introduced in Supplementary Material Fig. 1.

This section based on color analysis generally supports correlations and processes related to iron vs. calcium proposed in the literature^34,39^. However, the role of Fe might be more complex influencing not only calcium-containing aluminosilicates. Wu wrote in ref.^21^: “The Fe^2+^ spread rapidly in the brick bodies, and destroyed the crystal lattice of kaolinite and mica. Therefore, the decomposition temperature of kaolinite and mica could reduce 100–150 °C, and liquid phase in the brick bodies during the firing process would occur at a lower temperature”. The introduction of the additional a*/b* criterion seems to match well color changes and the processes related (only) to red hematite formation. Nevertheless, the discussion is around ’hematite’ with different colors and the correlation with Mossbauer data showing other iron oxides and Fe inside aluminosilicates requires more research and refinement. Therefore, the term hematite used in the color analysis should be viewed as defining all the hematite equivalent compounds, including the defected magnetite. Another uncertainty is related to non-uniform burning and color degradation as noted for example in Roman fired bricks from the Galerius Palace in Thessaloniki, Greece (fourth century AD)^19^. Color degradation was not investigated in our colorimetric study (superficial layer of the brick was removed). We mentioned a non-uniformity in color (see the first paragraph in this section) of the burnt brick B from Romula, but it is reasonably low. Due to this, we consider that it was mainly induced by non-uniform burning (the ratio a*/b* is 0.471 and 0.613 in regions B(L) and B(R), respectively, Table 4) rather than by degradation in the volume. Other symptoms of degradation on the surface or in the volume such as efflorescence of salts with and without scales, the presence of flakes and fungus on the surface, pulverization and loss of mass were at low level or lacking.

### Microstructural aspects of the burnt brick from Romula

**Figure 13:**
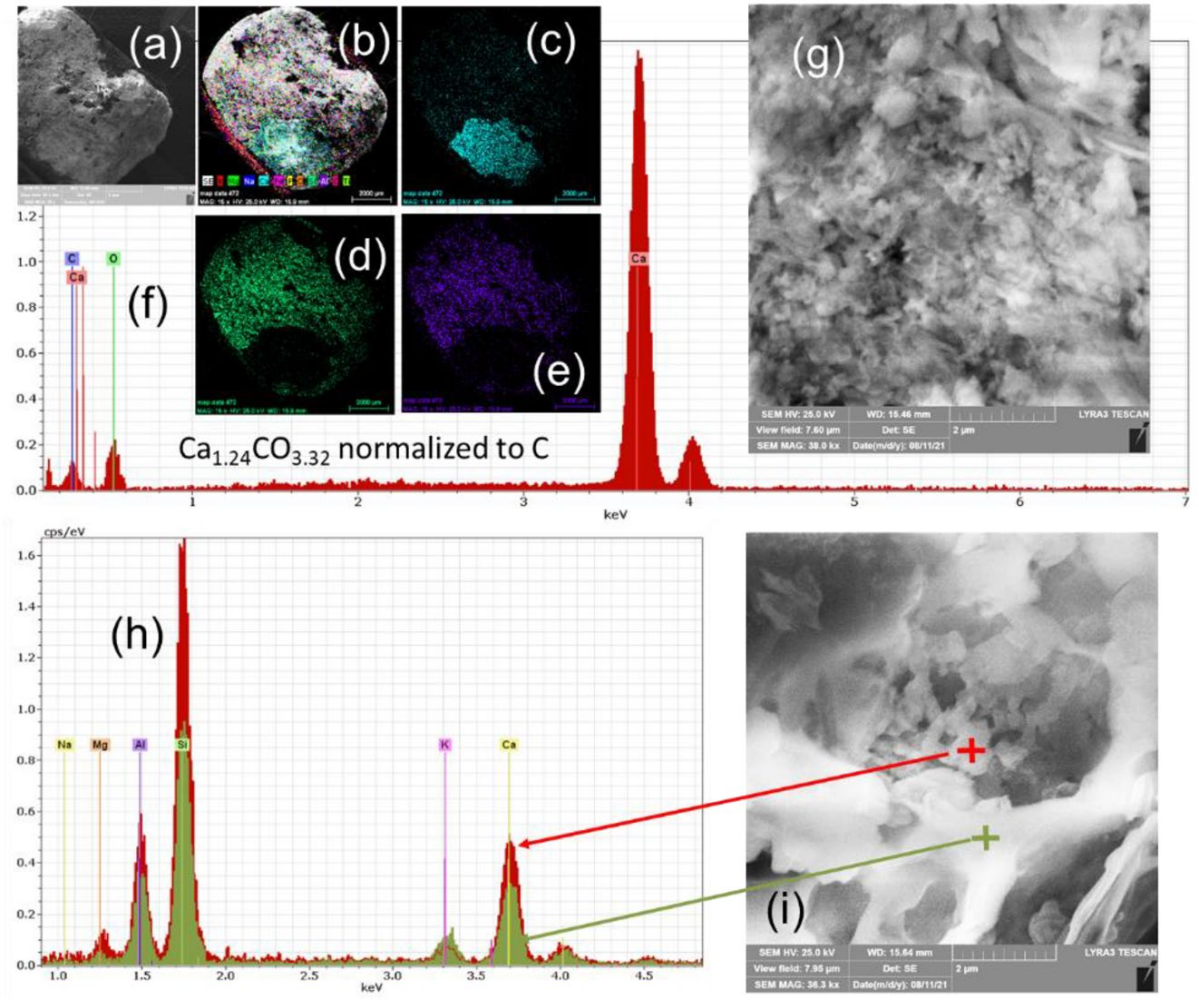
SEM image (**a**) and elemental maps for: (**c**) Ca; (**d**) O; (**e**) C. In (**b**) is presented RGB image obtained by overlapping the maps for individual elements; (**f**) EDX spectrum taken on the Ca-rich grain; (**g**) SEM image at high magnification showing nanostructuring of the Ca-rich grain; (**h**) EDX spectra taken inside and outside a pore shown in (**i**).

The microstructure of the burnt brick B presents non-uniformities. Not only Fe showed agglomeration, as already noted, but also Ca, Na, Mg, Al, K (Fig. 12) and Ti (not shown here). In the case of calcium, few large particles were found (< 4 mm) containing this element. An example is presented in Fig. 13a–e. According to EDX data, although this method is unreliable for quantitative evaluation of light elements such as C and O, the composition is Ca_1.24_CO_3.32_ (normalized to C) (Fig. 13g) and it can be written as (CaCO_3_)(CaO)_0.24_O_0.08._ Therefore, the Ca-rich large particle is mainly composed of CaCO_3_ and some CaO. Its appearance at high magnification (Fig. 13) is of an agglomerate composed of small nanoparticles (50–300 nm) and of few flake-like crystals in the micron range. Assuming that during burning of the Roman brick the initial CaCO_3_ from the soil entirely decomposed and formed CaO, the presence of CaCO_3_ nanoparticles in the brick of ~ 2000 years old suggest reformation of this phase in the presence of CO_2_ from the air. Reactions and their implications are discussed in “Durability aspects of the Roman bricks”.

Systematically, in the pores, nanoparticles (200–400 nm) were observed. EDX indicated that they have a higher content of Ca element (Fig. 13h,i) than outside the pore and were accompanied by a higher amount of Mg and Fe and a lower amount of K. This observation agrees with the Ca and Fe interaction proposed by Maniatis et al.^35^ (see “Colorimetric analysis of the soils and bricks from Romula”).

The morphology of the nanoparticles in the pores resembles that of the CaCO_3_ particles in Fig. 13g, but it was impossible to ascribe a phase because when measuring small particles comparable with the EDX spot size, the influence of the surroundings is high and results on measured compositions can be misleading. Detection of light elements by EDS is another problem, as already mentioned.

SEM observations at different magnifications (Supplementary material Fig. 2) on the burnt brick B from Romula provided no clear evidence for the presence of a glassy phase as proof for fusion or vitrification. No cracks were found. Some grains show a plate like hexagonal morphology typical for raw clays. These characteristics suggest that even though a glassy phase has appeared, its amount has been low. On the other side, for example the phase diagram of K_2_O–Al_2_O_3_–SiO_2_^37^ show the presence of liquid phases below 800 °C. A ternary eutectic forms at 695 °C.

## Discussion

### Burning temperature of the Roman brick from Romula

Following the reported results from ref.^35^, microstructural details from “Microstructural aspects of the burnt brick from Romula”, the information that CO_2_ release in the heated soil DS1 from Romula ends at 802 °C (TG and mass spectroscopy results, Fig. 5), and the colorimetric-Mӧssbauer correlations from “Colorimetric analysis of the soils and bricks from Romula” agree well with a burning temperature for the Ca-rich clays of ~ 850 °C. In the Ca-rich clays a glass phase or new aluminosilicates (e.g. cristobalite, mullite) are found in bricks burnt above 900 °C^38^. This is not our case. At high temperatures (> 900 °C) Fe-phases (hyrcite and hematite) occur, but these phases also were not detected by XRD in the burnt brick from Romula. Hence, all results indicate a firing temperature of 800–850 °C for the investigated burnt brick B from Romula.

**Figure 14:**
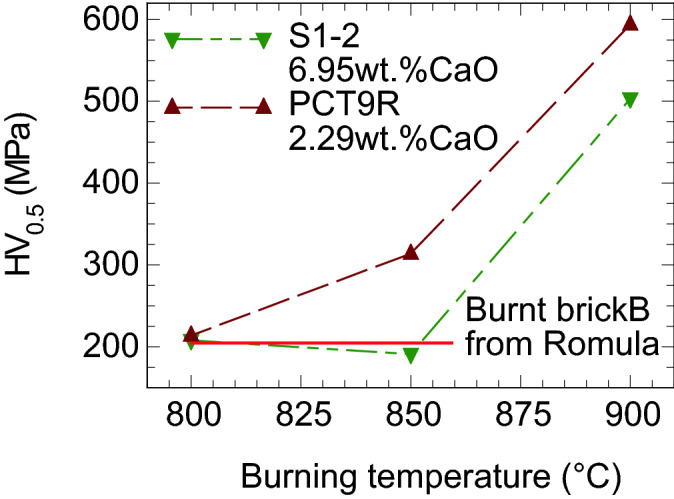
Vickers hardness measured on burnt brick B and ceramic samples manufactured in the lab from the as-excavated soil PCT9R with low content of calcium and from the mud brick (S1–2) rich in calcium.

Additional supporting arguments are based on comparison of Vickers hardness (Fig. 14) measured on the clay region of the burnt brick B and on small burnt cylinder samples (10 mm diameter and ~ 8 mm height) sintered in the laboratory from the as-excavated soil (PCT9R) and the mudbrick (S1–2). The value of HV_0.5_ measured on the Roman burnt brick B was 201 ± 22 MPa. The lab-made ceramic samples fired at 800 °C and the Roman brick B show similar HV_0.5_. At 850 °C, ceramic sample made in the lab from calcium-rich S1–2 (XRF data in Table 2) has approximately the same HV_0.5_ as at 800 °C, whereas for the sample manufactured from calcium-poor (XRF data in Table 2) PCT9R, HV_0.5_ becomes higher, as expected. When burning is performed at 900 °C, both ceramic samples obtained in the lab from S1-2 and PCT9R attain at least twice higher HV_0.5_ values than for the Roman brick B.

Comparison of the colorimetric data (Table 4) between samples B and S1–2 burnt at 800 and 850 °C indicate a good matching especially between samples B and S1–2 burnt at 800 °C.

Mossbauer and magnetic investigations presented in “Local structure and magnetic properties of materials from Romula” have shown that in the sample S1–2* burnt in air at 880 °C for 1 h there are more hematite clusters, and their distribution is different comparative to sample B. All these details indicate that the Roman burnt brick B was heated at temperatures below 880 °C and for longer time than 1 h.

It is concluded that it is reasonable to consider a burning temperature around 800 °C or slightly higher, not exceeding 850 °C, for the studied Roman burnt brick (B) from Romula. Scalenghe et al. proposed a burning temperature of the Roman bricks they investigated^8^ of 750 °C for 14 h.

### Durability aspects of the Roman bricks

As already addressed in “Soils characterization and their identification as raw materials for fabrication of the ancient mud and burnt bricks” mud and burnt bricks from Romula contain a certain amount of Ca, which is higher in the mudbricks. The ratio Si/Ca (taken as SiO_2_/CaO, XRF data, Table 2) has an average value of ~ 30, 8, and 5.2 for the low-depth soils (≤ 1.5 m), burnt brick, and mudbrick, respectively. The values in the high-depth soils are ~ 5.4 and 1.1 for DS1 (2.5 m) and DS2 (3 m). The values of Si/Ca (taken as SiO_2_/CaO, XRF data, Table 2) for the mud brick and DS1 soil match well. Other details presented in “Soils characterization and their identification as raw materials for fabrication of the ancient mud and burnt bricks” also indicate a high level of resemblance between the DS1 soil and the mud brick. If mud brick is fabricated from DS1 soil, the following question is formulated: why high-depth soils for fabrication of mudbricks are used instead of low-depth ones that are easier extracted? This question leads to idea that in the production of mudbricks a certain amount of calcium, relatively high, was intentionally designed and introduced by using selected soils. Soils from Romula contain calcium as calcite or limestone (XRD data in Fig. 3, Supplementary material Table 3). This mineral plays an important role in geological processes and building technology. Its interaction with water saturated with CO_2_ or with heat generates products such as calcium bicarbonate (Ca(HCO_3_)_2_) and oxide (CaO), respectively (Supplementary material Table 6). The reaction to form bicarbonate is reversible. The oxide CaO reacts with water and slacked lime (Ca(OH)_2_) forms. This product further reacts with CO_2_ and the limestone is restored. This cycle of reversible reactions due to CaCO_3_ can ensure the excellent durability of the studied mud and burnt bricks. Some aspects and arguments to consider are:

(i) Calcium carbonate has a very low solubility in pure water (15 mg/L at 25 °C), but its solubility increases as the temperature of water decreases^44^. In the rainwater saturated with carbon dioxide, its solubility also increases due to the formation of more soluble calcium bicarbonate. These features suggest that the use of mud bricks in a relatively cold climate as in Romula located in the north of the Roman Empire might not be accidental.
(ii) Frost resistance of limestone was investigated in ref.^22^ and it was shown to be a process that depends on pores structure and water distribution in the pores: a degree of maximum 60% water saturation was found to ensure high frost resistance if large pores are not available. In the burnt brick water absorption is 24.9%, i.e. it is two times lower, and the amount of large pores is low (~ 3 vol.%, “Porosity, water absorption, mechanical and thermal insulating properties of the burnt brick from Romula”), but a direct comparison is not possible and further research is needed.
(iii) Reaction of lime (Ca(OH)_2_) with water is accompanied by an increase of 35% in weight and, more interesting, by 12% rise in volume^40^. By this effect, pores can be generated^41^, but they can be also cemented^42–44^. By extrapolation, cracks induced by processing, weathering, earth movement and earthquakes, or because of the impacts from the battles can self-heal. Similar reactions with water and heat as for calcite were proposed for dolomite (CaMg(CO_3_)_2 (s)_) (Supplementary material Table 6)^43^. In this case, the recarbonation reaction is different with formation of hydromagnesite (portlandite, Mg_5_(CO_3_)_4_(OH)_2_·4H_2_O). This mineral is known to occupy pores previously occupied by CaO and through crystallization pressure to induce radial cracks around the original carbonate grains^45–49^. Considering also that MgO hydrates very slowly^43^ and possibly does not keep up with seasonal variations in climate makes Mg less desirable in bricks and explains the low content used (XRF data, Table 2).
(iv) Mass spectroscopy results for high depth soils DS1 and DS2 in Fig. 5e,f shows occurrence of OH radical at low temperatures (Supplementary material Table 5). The origin can be Ca(OH)_2_, but also mica and chlorite. Lime was widely used by romans in constructions of buildings being the basic and most common binding material^50^ in mortars and plasters. Intentional use of lime in roman mud bricks is not documented. The presence of Ca(OH)_2_ in the mud brick should be demonstrated, being at the speculation level in the present work. During heating (< 200 °C) mica releases OH^51^ and (00 l) peaks in XRD are shifting, an effect that was observed when comparing our soil and mud brick samples before (PCT9R, S1–2) and after heating at 880 °C (PCT9R*, S1–2*, B). The loss and intake of OH in mica is reversible and it is accompanied by crystal lattice expansion or shrinkage^19^. Perhaps this mechanism can also contribute the Roman bricks to adapt to changing environmental conditions.
(v) Low-depth soils and burnt brick (B) show similar values of Si (represented as SiO_2_, XRF data in Table 2), while Si/Ca (i.e. SiO_2_/CaO ratio, XRF data in Table 2) is high (~ 30) in the low-depth soils comparative to the burnt brick. It results that addition of a Ca-rich material to the low-depth soil, or of sand to high-depth calcium-rich soils (DS1, DS2) is required. This result points to compositional control of the raw materials used for production of the burnt bricks. Addition of sand to the clay in different proportions for the fabrication of Roman bricks from approximately the same period is noted in ref.^19^. Authors of this ref. explain the motivation behind the sand addition as follows: (a) the presence of sand reduces the early cracking due to shrinkage during drying; (b) sand promotes shape stability of the brick; and (c) the feldspars contained by sand due to their low melting point promote sintering through formation at relatively low temperatures of a glassy phase. Our data suggest that sand addition was also aimed to control calcium and the calcium carbonate amount. A sand deposit was identified at Romula (Fig. 1b, inset) and a sample (S) was investigated (Table 1). FT-IR, XRF, and XRD results are presented in Fig. 4, Table 2, and Fig. 3, Supplementary material Table 3. Sand (S) is composed of minerals found in soils and in the mud brick. It has a low concentration of calcium in XRF (represented as oxide, Table 2) and the amount of carbonate phases (denoted Ca carbonate and Ca-Mg carbonate in XRD data from Fig. 3 and Supplementary material Table 3) is also low as for low-depth soils. At the same time, sand (S) shows the highest values for Si in XRF (as SiO_2_ oxide, Table 2) and of quartz in XRD (Fig. 3, Supplementary material Table 3) among all investigated samples. We appreciate that the sand (S) can be used as an additive to high-depth soils in the manufacturing of the burnt bricks from Romula to control and obtain the brick’s optimum recipe for excellent durability and outstanding mechanical properties (see “Mechanical response aspects of the Roman burnt brick from Romula”). A high amount of calcite during burning produces much CO_2_ and this impedes sintering, many pores are formed impacting quality^52,53^. A low amount of Ca would generate a high amount of glassy phase^35^ influencing the pores structure and interaction with environment. Mass spectroscopy and DTA/TG measurements indicate (Fig. 5, and Supplementary Material Table 5) that there are two steps of CO_2_ release. First step at low temperatures (~ 400 °C) is weak, while the second one at high temperatures (~ 800 °C) is intense. However, the amount of calcite in the sample, the presence of the other phases, and heating conditions modify the temperature and amplitude of the thermal effect. This indicates that the effect of calcite decomposition should be correlated with other factors of material features and processing.

This section discussed arguments for possible self-healing due to reversible phase changes under an external stimulus, e.g. humidity, CO_2_, and temperature variations accompanied by pores and cracks modification (local shape changes) thus providing certain responsiveness and adaptability of both mud and burnt bricks to external environmental changing conditions. Interaction of these materials is autonomous and as in the living organisms. These features are specific for modern advanced ’smart’ or ’intelligent’ materials^54^. This bold concept ascribed to ancient bricks is, in the authors’ view, needed to explain the millennium long durability of the ancient construction materials, but additional proofs and experimental demonstration are required. It is also suggested that weathering information must be expanded beyond degradation aspects. Our analysis also leads to idea that Romans were aware of the raw materials compositional control and optimization for obtaining bricks with excellent durability (see also “Mechanical response aspects of the Roman burnt brick from Romula”).

### Mechanical response aspects of the Roman burnt brick from Romula

Bricks with elastic modulus determined from standard compressive tests around 500 MPa, i.e. comparable with the value for the burnt brick B from Romula, are labeled as very soft^55^. Simultaneously, the burnt brick from Romula is also large. Large size bricks are justified by high productivity needs and fast building of large constructions^21,50^ such as the defense walls at Romula. The flat shape of the sesquipedalian Roman burnt bricks (~ 45 cm length, *L* and ~ 30 cm width, *W*) with small thickness (6 cm or in some cases down to 2 cm, *T*)^19^ is explained by easier drying and more uniform heating necessary for shape stability and to avoid cracks formation. The equivalent square size would be *a* = (L × W)^0.5^ = 36.74 cm. The shape factor *δ* = *T*/*a* = 6/36.74 = 0.16. For the ancient largest ever burnt brick excavated from Nanyue Kingdom Palace (202 BC–9 BC, Western Han Dinasty, Guangzhou, China^21^ measuring 100 cm × 40 cm × 20 cm the shape factor is 0.31. The Chinese brick has two times higher *δ* than for the Roman brick, but it is still significantly lower than 1, and its thickness is high, about 3.3 times larger than for the Roman brick which would make drying difficult and productivity would be limited. The humid hot weather in Guangzhou is acknowledged for successful drying without cracks of the large Chinese bricks^21^. In Romula weather is dry. Processing specifics influenced by the local climate are of primary concern, but might not be the only factors that are involved in the design of the bricks. The shape factor influences compressive behavior of the bricks^23,56^:

3 $$\sigma_{{\text{n}}} = c \times \delta \times \sigma_{{1}}$$

where *σ*_n_ is the normalized compressive strength, *c* is conditioning factor (*c* = 1), and *σ*_1_ is the measured compressive strength (at the first crack). The blue curve in Fig. 7 was measured on a sample cut from the Roman burnt brick B with dimensions (2.3 cm × 1.5 cm × 0.3 cm) at 1:20 scale of the original brick size. Introducing in formula (3) *δ* = 0.16 and *σ*_1_ = *σ*_1_^1:20^ = 19 MPa (Fig. 7, blue curve), *σ*_n_ is 3.1 MPa. This value is acceptable, but somehow lower than the value of 5.6 MPa measured on standard cubic samples.

More interesting is the extraordinary behavior of the 1:20 sample (burnt brick B) under compressive load as revealed by the compressive stress–strain curve (Fig. 7, blue curve) and optical microscopy images taken on the sample before attaining *σ*_1_^1:20^, at *σ*_2_^1:20^ and at *σ*_3_^1:20^ = 350 MPa (photos 1–3, Fig. 7). When the brick sample is subject to an increasing force in the 0-*σ*_1_^1:20^ range, the response is of elastic type until the first macrocrack occurs (see optical microscopy photo 2, Fig. 7). The cracks initiate on the edges and corners that act as stress concentrators as shown also by FEM simulation (Fig. 8). Macrocracks propagate, but although load increases, they do not reach the center of the sample and do not connect so that the integrity of the brick sample is preserved except the corners. Thickness reduction at *σ*_3_^1:20^ was 36.25%. This value is comparable with the porosity of the brick, *P* = 39.4% suggesting that most pores were removed at *σ*_3_^1:20^. From *σ*_1_^1:20^ to *σ*_2_^1:20^ plastic deformation is high, and it is due to local gradual fracturing of the material with removal of the pores, but sustaining the compressive load. Above *σ*_2_^1:20^ the material behaves as a typical compressed powder with fewer pores so that the compressive strength rapidly increases, and the sample becomes a powder compact. Apart from convenient shape with low shape factors, the microstructure with small and distributed pores (< 1 µm), lack of brittle glassy phases, composite effects where clay provides a network (matrix) in which sand and other phases are embedded are thought also to play an important role in observed behavior.

Results on the 1:20 brick sample may suggest that ancient brickmakers were aware of the importance of the shape and they performed some optimization in this regard. The shape and specific material give the burnt bricks unexpected adaptability to compressive load preserving their integrity.

## Conclusion

This work presents a complex archaeometric characterization of the Roman sesquipedalian bricks of mud and burnt type from Romula. To authors knowledge, this is the first study of its kind on the Roman bricks from the Lower Danube provinces of the Roman Empire. Excavation of mud bricks in these northern Roman provinces is an unexpected discovery and deserves further attention. Possible raw materials, mudbricks, and burnt bricks are compared.

Results indicate useful correlations between different properties also drawing attention on limitations and the need for refinements. Data are valuable towards the development of future improved forensic methods, archeological progress, conservation/restoration solutions and as reference to compare with other ancient and modern ceramic materials. Work shows that in some cases, comparison of properties without understanding the physical background can be misleading: when e.g. colors are assessed or magnetic properties are analyzed and compared, understanding of the compositional, structural, microstructural, and fine structure details is needed. In other words, very different artefacts can have apparently similar magnetic or colorimetric properties and a direct comparison is not enough to draw conclusions.

Our analysis suggests that Roman bricks are responsive, can adapt to external factors, and can be included to a certain degree within the modern concept of ’smart/intelligent’ materials. These special features can explain excellent durability and long-life function of the Roman bricks. Some evidence promotes the idea that Roman brickmakers implemented raw materials and recipe control, selection of the optimum technological parameters to achieve the desired physical–chemical–mechanical properties, and more surprising, they empirically applied microstructure, size, and shape design. More research is needed to support our claims.

The criteria for the fabrication of durable Roman bricks can be summarized as follows:

(i) Raw materials for fabrication of mud and burnt bricks are a mixture of ~ 40 wt.% clay and 60 wt.% sand with a relatively small particle size (< 2 mm).
(ii) The amount of alkali metals such as Na, K should be relatively low, ~ (3–5) wt.% (oxide representation). This is required to avoid excessive melting during thermal processing and, thus, to limit the formation of a high amount of the hard, but brittle glassy phase. The glassy phase impacts microstructure (pores size and distribution) and further the weathering and mechanical behavior of the burnt brick.
(iii) Separation of Fe from the aluminosilicates, clusterization, and oxidation were revealed. Iron has a complex behavior and role in controlling the stability and decomposition of silicates. Its interplay with calcium is especially important, but more research is needed to understand the details considering each silicate type and mixtures. The relative amount of Fe (represented as oxide in XRF results) takes values in the 6–10 wt.% range.
(iv) The amount of calcium is relatively high in both mud and burnt bricks from Romula. For the burnt brick, calcium represented as oxide in XRF is around 6–8 wt.%, while in the mudbrick it is even higher, around 15–19 wt.%. The amount of calcium (and of calcium carbonate in particular) in the bricks should be optimum to contribute pores development or, more general, the microstructure formation and control during processing (drying and burning). Calcium plays a key role in the reversible long-term chemical processes of the brick interaction with the environment with a strong effect on cracks and pores behavior. Other mechanisms of the brick-environment interaction should also consider silicates crystal chemistry during processing and during their long-term use.
(v) The amount of Mg (represented as oxide in XRF results) in mud and burnt bricks should be relatively low (1–2 wt.%), significantly lower than that of calcium. A high amount negatively impacts cracks and pores behavior during processing and long-term interaction of the brick with the environment.
(vi) The shape and size of the burnt brick, the small distributed pores and reinforcement particles (large pores or particles are only about 3–3.5 vol.%) in the aluminosilicates matrix and the lack of a high amount of brittle glassy phase in the matrix are essential for mechanical properties. Although these burnt bricks are classified as soft, they can withstand large compressive mechanical loads, and more important, large load variations, adapting and preserving their integrity.
(vii) Burning temperature of the bricks is optimized to ensure previous criteria. For the burnt brick from Romula the temperature is slightly above 800 °C and below 850 °C.

## Supplementary Information

Supplementary Figure 1.

Supplementary Figure 2.

Supplementary Table 1.

Supplementary Table 2.

Supplementary Table 3.

Supplementary Table 4.

Supplementary Table 5.

Supplementary Table 6.

## Data Availability

The datasets used and/or analysed during the current study are available from the corresponding author on reasonable request.
