# Supplementary material for: Mud and burnt Roman bricks from Romula
Source: Sci Rep. 2022 Sep 23;12:15864. doi: 10.1038/s41598-022-19427-7 (PMC9508116; doi:10.1038/s41598-022-19427-7)
Supplement: Supplementary file 1 — Supplementary Figure 1. [file 41598_2022_19427_MOESM1_ESM.docx]

| 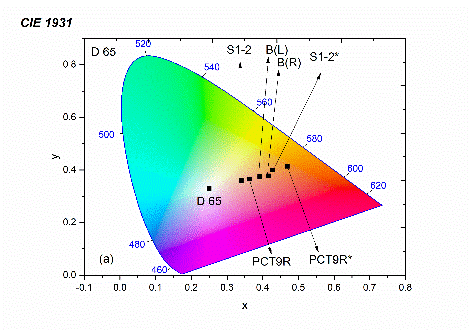 | 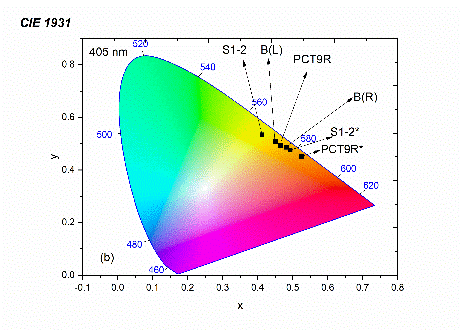 | 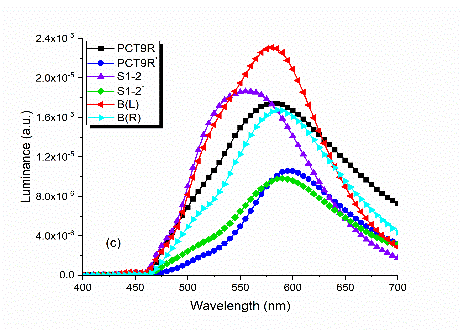 |
| --- | --- | --- |
|  |  |  |

**Supplementary material Fig. 1** (a)- CIE representation of the reflected D65 light for investigated materials from Romula; (b)- CIE representation under 405 nm light; (c)- Reflection spectra under 405 nm light. B(L) and B(R) indicates 2 different locations where colorimetric measurements were performed on the burnt brick B from Romula.

Note the differences between data for D65 in (a) and 405 nm in (b) illumination due to absorption of the blue light. However, the CIE xy representation and the recorded spectra under 405 nm illumination exhibit the expected redshift between the initial and the respective thermally treated samples. The shift might be assigned to gradual conversion of the initial clay with defected hematite towards pure hematite structures with the annealing temperature. For example, burnt sample PCT9R* after annealing at 880 °C reveals a redshift from 583 nm (in PCT9R) to 596 nm, while for S1-2 / S1-2* the redshift is from 555 nm to 590 nm. In the case of the burnt brick B, the small redshift value from 580 nm to 584 nm, indicates a less defected hematite, the shift being due to annealing temperature gradient during the burning process. A larger redshift in the mudbrick with a larger amount of calcium (oxide representation in XRF Table 2) than for the soil is somehow contrary to expectations, and deserves further attention. An explanation is currently missing. We also observe the different shape of the curves.

The differences between the reflections spectra measured at D65 illuminations and those obtained under 405 nm irradiation, indicate: (a) under D65 illumination, the peak reflections are around 580 nm, meaning that absorption is low; (b) under 405 nm irradiations, the peak reflections are spread between 568 nm to 583 nm due to a higher absorption of the UV light by pigments; the effect is relevant especially for defected hematite.
