# Supplementary material for: Mud and burnt Roman bricks from Romula
Source: Sci Rep. 2022 Sep 23;12:15864. doi: 10.1038/s41598-022-19427-7 (PMC9508116; doi:10.1038/s41598-022-19427-7)
Supplement: Supplementary file 2 — Supplementary Figure 2. [file 41598_2022_19427_MOESM2_ESM.docx]

**
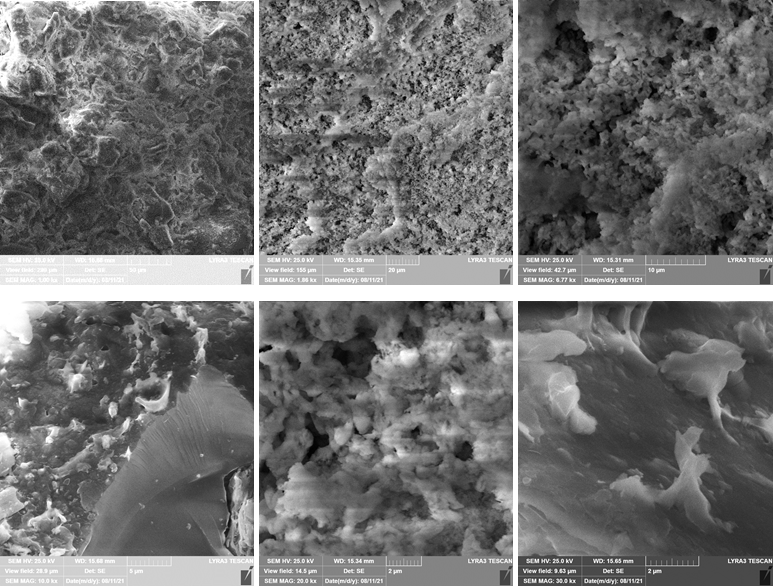
**

**Supplementary material Fig. 2** SEM images taken on the Roman brick from Romula (sample B) for increasing magnification.
