# Supplementary material for: Mud and burnt Roman bricks from Romula
Source: Sci Rep. 2022 Sep 23;12:15864. doi: 10.1038/s41598-022-19427-7 (PMC9508116; doi:10.1038/s41598-022-19427-7)
Supplement: Supplementary file 3 — Supplementary Table 1. [file 41598_2022_19427_MOESM3_ESM.docx]

**Supplementary material Table 1**. Methodology and equations to determine different parameters of the Roman burnt brick from Romula by Archimedes approach according to *C134* and *C830* ASTM standards.

| **No.** | **Methodology** | **Formula** |
| --- | --- | --- |
| 1 | A sample of 20 mm length, 20 mm width and 20 mm thickness cut from the burnt brick in Fig. 2a has been weighted (*A* = dry weight) after drying to 110 ^o^C for 4 h. | - |
| 2 | After cooling to room temperature, the sample, while being in the water and suspended, was weighed (*B* = suspended weight in the water). The sample was removed from the container with water and, after wiping the water from the surface it was weighted in the air to obtain de water-saturated weight *C*. The exterior volume of the sample, considering that 1 cm^3^ of water weights 1g, was determined: | $V \left( {cm}^{3} \right)=C-B$ (1) |
| 3 | The volume of the open pores and of the impervious part were calculated: | $V open pores \left( {cm}^{3} \right)=C-A$ (2)  $V impervious part \left( {cm}^{3} \right)=A-B$ (3) |
| 4 | The apparent porosity is the ratio between the volume of the open pores in the sample to its exterior volume: | $P\left( \% \right)=[(C-A)/V]\times100$ (4) |
| 5 | The water absorption *Abs* is the weight of water absorbed reduced to the dry weight of the sample: | $Abs\left( \% \right)=[(C-A)/A] \times100$ (5) |
| 6 | The bulk density *ρ* of the sample is the result of its dry weight divided by the exterior volume, including pores: | $(g/{cm}^{3})=A/V$ (6) |
| 7 | The bulk density of the material, without open pores is: | $R(g/{cm}^{3})=A/(A-B)$ (7) |
