# Supplementary material for: Mud and burnt Roman bricks from Romula
Source: Sci Rep. 2022 Sep 23;12:15864. doi: 10.1038/s41598-022-19427-7 (PMC9508116; doi:10.1038/s41598-022-19427-7)
Supplement: Supplementary file 4 — Supplementary Table 2. [file 41598_2022_19427_MOESM4_ESM.docx]

**Supplementary material Table 2**. X-ray computer tomography (XCT) experimental arrangement and measurement details.

| **No.** | **Details of XCT arrangement, measurement, and analysis** |
| --- | --- |
| 1 | The computer tomograph (CT) configuration includes a nanofocus - X-ray tube that can operate at a voltage up to 225 kV and a maximum target power of 30 W. |
| 2 | The radiographic images were acquired with a Dexela CMOS flat panel composed of 1944 x 1536 pixels, each with a size of 74 μm, placed at a distance at 584 mm from the X-Ray source. |
| 3 | The sample was scanned at a voltage of 80 kV and a current of 300 μA using 1 mm Al filtration and placed at 66 mm from the X-ray source. A total of 1200 projection were acquired at an incremental step on 0.3 degree. To provide a good signal to noise ratio, two frames were averaged before each projection storage at an integration time of 1 sec. |
| 4 | After data acquisition, the 3D model was reconstructed with Volume Graphics Software (VGStudio Max 3.3.0) using the cone-beam CT reconstruction module and filtered back-projection algorithm. A nominal voxel resolution of 7.5 μm was obtained for the reconstructed model. |
| 5 | The detection of pores and inclusions was performed using the dedicated module Porosity/Inclusions Analysis from the same VGStudio Max software. Surface determination was performed before the porosity analysis to separate the edges of the sample from the surrounding air. An advanced function for the surface determination was used with special numerical treatments like “use healing parameter”, and “remove all particles and voids”. |
| 6 | Next, a 3D box was defined as the region of interest over the total reconstructed volume of the sample and extracted for the porosity and inclusions analysis. The pore/inclusion detection was performed using a threshold type algorithm. The optimum threshold values were determined manually by reading the grey value inside the pores or inclusions from one cross sectional view and using the preview function available in the Porosity/Inclusions Analysis menu. The algorithm was set to detect all pores and inclusions with volumes in the range from 16 voxels up to full characteristic size of the sample. |
