# Supplementary material for: Mud and burnt Roman bricks from Romula
Source: Sci Rep. 2022 Sep 23;12:15864. doi: 10.1038/s41598-022-19427-7 (PMC9508116; doi:10.1038/s41598-022-19427-7)
Supplement: Supplementary file 5 — Supplementary Table 3. [file 41598_2022_19427_MOESM5_ESM.docx]

**Supplementary material Table 3.**  Phase amount determined from XRD (Fig. 3) by Rietveld analysis. Sample notation is as in Table 1.

| **Sample** | **Quartz (Q)**  **(wt. %)** | **Chlorite (Ch)**  **(wt. %)** | **Mica (M)**  **(wt. %)** | **K-feldspar**  **(F)**  **(wt. %)** | **Plagioclase**  **(P)**  **(wt. %)** | **Ferri-tschermakite**  **(T)**  **(wt. %)** | **Calcite**  **(Ca carbonate)**  **(wt. %)** | **Calcite with Mg**  **(Ca-Mg carbonate)**  **(wt. %)** |
| --- | --- | --- | --- | --- | --- | --- | --- | --- |
| PCT9R* | 27.4 | <1 | 27.4 | 5.8 | 13.6 | 24.6 | <1 | <1 |
| PCT9R | 26.9 | <1 | 32.9 | 4.8 | 10.5 | 23.7 | <1 | <1 |
| S1-2* | 34.6 | <1 | 20.2 | 2.8 | 21.1 | 20.4 | <1 | <1 |
| S1-2 | 31.1 | <1 | 20.6 | 8.5 | 13.3 | 24.2 | <1 | 1.1 |
| DS2 | 20.9 | <1 | 20.3 | <1 | 11.6 | 27.4 | 17.9 | 1.6 |
| DS1 | 24.1 | <1 | 29.5 | <1 | 19.4 | 23.8 | 2 | <1 |
| S | 59 | <1 | 9.3 | 2.9 | 7.5 | 20 | <1 | <1 |
| B | 26 | <1 | 19.9 | 8.6 | 17.2 | 21.8 | 1.2 | 4.8 |

(*) – soil (PCT9R) and mud-brick (S1-2) powder samples: as-excavated and ignited in laboratory at 880 °C in air with a heating rate of 200 °C/h, for a dwell time of 1 h.
