# Supplementary material for: Mud and burnt Roman bricks from Romula
Source: Sci Rep. 2022 Sep 23;12:15864. doi: 10.1038/s41598-022-19427-7 (PMC9508116; doi:10.1038/s41598-022-19427-7)
Supplement: Supplementary file 6 — Supplementary Table 4. [file 41598_2022_19427_MOESM6_ESM.docx]

**Supplementary material Table 4.** Identified minerals by XRD and FT-IR methods (see Figs. 3, 4 in the manuscript) considering international databases of powder diffraction files and refs. [1-4].

| **Mineral** | **Chemical formula / density**  **(g/cm^3^)** | **XRD peaks used for phase identification**  **2θ (°)** | **FT-IR peaks used for phase identification (cm^-1^)** | **Ref.**  **XRD** | **Ref.**  **FT-IR** |
| --- | --- | --- | --- | --- | --- |
| Quartz  (Q) | α-SiO_2_  2.76 | 20.83; 26.67; 36.50; 39.49; 40.29; 42.42; 45.83; 50.17 | 773; 1087; 1155 | [1, 2] | [3, 4] |
| Calcite  (Ca carbonate) | α-CaCO_3_  2.71 | 25.5; 29.5; 36.1; 43.3; 48.7 | 708; 875; 1408 | [2] | [3, 4] |
| Calcite magnesian  (Ca-Mg carbonate) | (Ca, Mg)CO_3_  2.73 | 29.71; 36.3; 48.12 | 876;1470 | [2] | [3, 4] |
| Ferri-tschermakite  (T) | Na_0.5_Ca_1.8_Mg_2.2_Ti_0.1_Fe_2_Al_2.4_Si_6.4_  O_22.2_(OH)_1.8_  3.22 | 10.53; 19.65; 48.22;48.73 | 984; 1075 | [2] | [3, 4] |
| Chlorite  (Ch) | (Mg_,_Al)_6_(Al,Si)_4_O_10_(OH)_8_  2.67 | 12.40; 19.76; 25.14; 34.73; 35.02 | 650; 1008; 3400-3700 | [1, 2] | [3, 4] |
| Mica  (Muscovite)  (M) | KAl_2_(AlSi_3_O_10_)(OH)_2_  2.83 | 8.88; 17.85; 19.87; 35.02; 45.5 | 520; 1023; 3400-3650 | [1, 2] | [3, 4] |
| Plagioclase  (P) | (Na,Ca)[(Si,Al)_4_O_8_  2.67 | 13.91; 22.03; 27.95; 30.56; | 600; 1005; 1097 | [1, 2] | [3, 4] |
| Feldspar  (K-spar)  (F) | KAlSi_3_O_8_  2.56 | 27.53 | 1011; 1055; 1143 | [1, 2] | [3, 4] |

**Cross References:**

[1] Scalenghe, R. Material sources of the Roman brick-making industry in the I and II century A.D. from Regio IX, Regio XI and Alpes Cottiae. *Quaternary International* 356, pp. 189-206 (2015).

[2] Aslan, O. Properties of Roman Bricks and Mortars used in Serapis Temple in Bergama*. Izmir Institute of Technology* (2005).

[3] Moore, M., Reynolds, R.C. X-ray Diffraction and the Identification and Analysis of Clay Minerals. *Oxford University Press* (1997).

[4] Chukanov, N. Infrared spectra of minerals species. *Springer Geochemistry/Mineralogy* (2014).
