# Supplementary material for: Mud and burnt Roman bricks from Romula
Source: Sci Rep. 2022 Sep 23;12:15864. doi: 10.1038/s41598-022-19427-7 (PMC9508116; doi:10.1038/s41598-022-19427-7)
Supplement: Supplementary file 7 — Supplementary Table 5. [file 41598_2022_19427_MOESM7_ESM.docx]

**Supplementary material Table 5.** Reaction/decomposition stages (I - RT-180 ^o^C; II - 180-400 ^o^C; III- 400-878 ^o^C; IV- 878-1100 ^o^C) observed in thermal analysis DTA/TG/mass spectroscopy experiments (see Fig. 5) performed in air with a heating rate of 10°C/min. Samples notation is as in Table 1. Processes during heating and phase formation were indicated considering literature [1-3].

| **Sample** | **Stage** | **Type** | **DSC** | | **Weight variation (%)** | **Processes during heating and phase formation** | **References** |
| --- | --- | --- | --- | --- | --- | --- | --- |
|  |  |  | **T^onset^(^o^C)** | **T^offset^(^o^C)** |  |  |  |
| PCT9R | I | endo | 93 | 177 | 6.7 | Water and hydroxide loss | [1] |
|  | II | exo | 180 | 400 | 1.1 | Chlorite breakdown | [2] |
|  | III | endo | 485 | 525 | 3.5 | Carbon dioxide loss | [1] |
|  |  | endo | 565 | 580 | 0.1 | Quartz inversion | [3] |
| DS1 | I | endo | 55 | 100 | 0.5 | Water and hydroxide loss | [1] |
|  |  | endo | 109 | 160 | 0.7 |  |  |
|  | II | exo | 275 | 375 | 0.7 | Chlorite breakdown | [2] |
|  | III | endo | 470 | 550 | 0.8 | Carbon dioxide loss | [1] |
|  |  | endo | 567 | 588 | 0.1 | Quartz inversion | [3] |
|  |  | endo | 725 | 802 | 1.5 | Carbon dioxide loss and calcite breakdown | [1] |
|  | IV | exo | 880 | 925 | 0.02 | Plagioclase breakdown | [2] |
| DS2 | I | endo | 56 | 100 | 0.1 | Water and hydroxide loss | [1] |
|  |  | endo | 117 | 170 | 0.1 |  |  |
|  | II | exo | 303 | 356 | 0.03 | Chlorite breakdown | [3] |
|  | III | endo | 566 | 587 | 0.2 | Quartz inversion | [3] |
|  |  | endo | 740 | 865 | 7.2 | Carbon dioxide loss and calcite breakdown | [1] |
|  | IV | exo | 886 | 930 | 0.1 | Plagioclase breakdown | [2] |
| S1-2 | I | endo | 110 | 178 | 0.5 | Water and hydroxide loss | [1] |
|  | II | exo | 180 | 400 | 0.8 | Chlorite breakdown | [2] |
|  | III | endo | 567 | 580 | 0.6 | Quartz inversion | [3] |
|  | IV | exo | 878 | 1100 | 0.4 | Plagioclase breakdown | [2] |
| S1-2* | I | endo | 40 | 90 | 0.3 | Oxidation: Fe^2+^ → Fe^3+^ | See section 3.3 |
|  |  | endo | 126 | 178 | 0.06 |  |  |
|  | II | exo | 352 | 372 | 0.01 | Chlorite breakdown | [2] |
|  | III | endo | 400 | 433 | 0.1 |  |  |
|  |  | endo | 569 | 588 | 0.03 | Quartz inversion | [3] |
|  | IV | exo | 893 | 913 | 0.01 | Plagioclase breakdown | [2] |
| B | I | endo | 40 | 114 | 1 | Water and hydroxide loss | [1] |
|  |  | endo | 124 | 158 | 0.02 |  |  |
|  | II | endo | 198 | 238 | 0.2 | Chlorite breakdown | [2] |
|  |  | exo | 352 | 359 | 0.3 |  |  |
|  | III | endo | 568 | 586 | 0.03 | Quartz inversion | [3] |
|  |  | endo | 670 | 717 | 1.2 | Hematite crystallization and carbon dioxide loss | [2] |
|  | IV | exo | 878 | 1100 | 1 | Plagioclase breakdown | [2] |

**Cross References:**

[1] Scalenghe, R. Material sources of the Roman brick-making industry in the I and II century A.D. from Regio IX, Regio XI and Alpes Cottiae. *Quaternary International.* 356, pp. 189-206 (2015).

[2] Raman, C.V., Nedungadi, T.M. The α-β Transformation of Quartz. *Nature.* 145, pp. 147 (1940).

[3] Bellanger, M., Homand, F., Remy, J.M. Water behavior in limestones as a function of pores structure - application to frost-resistance of some lorraine limestones*. Engineering Geology.* 36, pp. 99–108 (1993).
