# Supplementary material for: Mud and burnt Roman bricks from Romula
Source: Sci Rep. 2022 Sep 23;12:15864. doi: 10.1038/s41598-022-19427-7 (PMC9508116; doi:10.1038/s41598-022-19427-7)
Supplement: Supplementary file 8 — Supplementary Table 6. [file 41598_2022_19427_MOESM8_ESM.docx]

**Supplementary material Table 6.** Reactions related to calcite and dolomite involved in Roman brick response to environment changes (humidity, temperature).

| **No.** | **Reaction** | **Observation** |
| --- | --- | --- |
| **1** | CaCO_3(s)_ + CO_2(g)_ +H_2_O_(l)_ ↔ Ca(HCO_3_)_2(aq)_ | - |
| **2** | CaCO_3 (s)_ + heat → CaO_(s)_ + CO_2 (g)_↑ | - |
| **3** | CaO_(s)_ +H_2_O_(l)_ → Ca(OH)_2 (s)_ + heat | - |
| **4** | Ca(OH)_2_ + CO_2_ → CaCO_3_ + H_2_O | Recarbonation reaction of calcite |
| **5** | CaMg(CO_3_)_2 (s)_ + heat → CaO_(s)_ + MgO_(s)_ + 2CO_2 (g)_↑ | - |
| **6** | MgO_(s)_ +H_2_O_(l)_ → Mg(OH)_2 (s)_ | - |
| **7** | 5Mg(OH)_2_ + 4CO_2_ → Mg_5_(CO_3_)_4_(OH)_2_·4H_2_O | The recarbonation reaction when considering dolomite is different than for calcite (No. 4) |
